# Diisoprenyl Cyclohexene-Type Meroterpenoids with Cytotoxic Activity from a Mangrove Endophytic Fungus *Aspergillus* sp. GXNU-Y85

**DOI:** 10.3390/md22020058

**Published:** 2024-01-24

**Authors:** Feng Qin, Zi-Shuo Song, Li Luo, Xiang-Long Bo, Fu-Rong Wu, Mei-Jing Tan, Fan-Fan Wang, Xi-Shan Huang, Heng-Shan Wang

**Affiliations:** 1 State Key Laboratory for Chemistry and Molecular Engineering of Medicinal Resources/Key Laboratory for Chemistry and Molecular Engineering of Medicinal Resources (Ministry of Education of China), Collaborative Innovation Center for Guangxi Ethnic Medicine, School of Chemistry and Pharmaceutical Sciences, Guangxi Normal University, Guilin 541004, China; qinf1114@163.com (F.Q.); zishuos@163.com (Z.-S.S.); luoli51598@163.com (L.L.); bxl4937@foxmail.com (X.-L.B.); xingkong2137@gmail.com (F.-R.W.); tanmeijing2022@163.com (M.-J.T.); 2 Guangxi Key Laboratory of Agricultural Resources Chemistry and Biotechnology, College of Chemistry and Food Science, Yulin Normal University, Yulin 537000, China; fanfanwang2020@163.com

**Keywords:** mangrove endophytic fungus, *Aspergillus* sp., meroterpenoids, anti-cancer activity

## Abstract

Five new diisoprenyl cyclohexene-type meroterpenoids, aspergienynes J–N (**1**–**5**), along with three known analogues (**6**–**8**), were obtained from the mangrove endophytic fungal strain *Aspergillus* sp. GXNU-Y85. The chemical structures, including their absolute configurations, were established via spectroscopic data and comparison of experimental and calculated ECD spectra. Cytotoxicity assay results indicated that compound **8** had strong cytotoxicity against HeLa cancer cells, and its IC_50_ value was 11.8 μM. In addition, flow cytometry analysis revealed that the cytotoxicity of **8** was due to the induction of G1 cell cycle arrest and apoptosis in HeLa cells.

## 1. Introduction

As prolific microbial resources, marine-derived endophytic fungi can produce many biologically active secondary metabolites, such as sesquiterpenes, alkaloids, and polyketides [1,2]. Previous research reports have examined diisoprenyl cyclohexene-type meroterpenoids, which exhibit various biological activities, including cytotoxic [3,4], anti-inflammatory [5], antimicrobial [6], anti-H1N1 viral [7], and anti-nonalcoholic steatohepatitis [8]. The fascinating structural and biological properties of diisoprenyl cyclohexene-type meroterpenoids make it possible for them to be developed into potential leading compounds for cancers from natural products [3].

**Figure 1:**
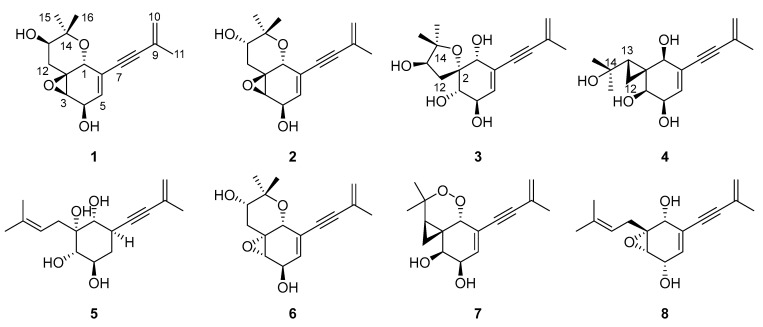
The chemical structures **1**–**8**.

As part of our continuing research to find mangrove endophytic fungi-derived bioactive constituents, the chemical constituents of *Aspergillus* sp. Y85 were investigated. The chemical investigation of the secondary metabolites of *Aspergillus* sp. Y85 from the fruit of *Kandelia candel*, a mangrove plant, resulted in the isolation of five undescribed diisoprenyl cyclohexene-type meroterpenoids, aspergienynes J–N (**1**–**5**), together with three previously reported analogues (**6**–**8**) (Figure 1). Compounds **1**–**8** were evaluated for their cytotoxic activity against five human cancer cell lines. Herein, the isolation, structure elucidation, and cytotoxicity of (**1**–**8**) are described.

## 2. Results and Discussion

**Figure 2:**
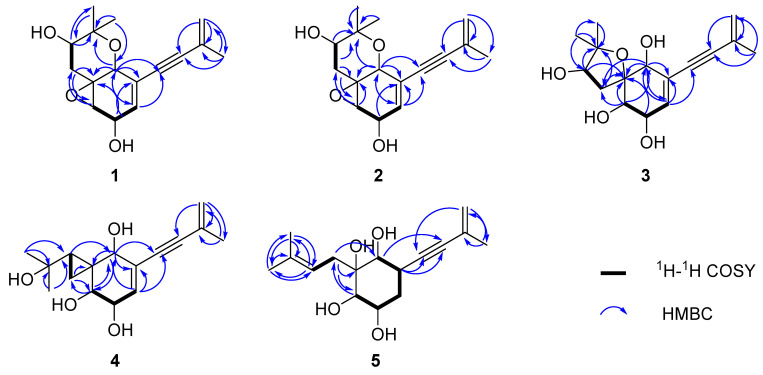
Key COSY (bold black line) and HMBC (blue line) correlations of compounds **1**–**5**.

**Figure 3:**
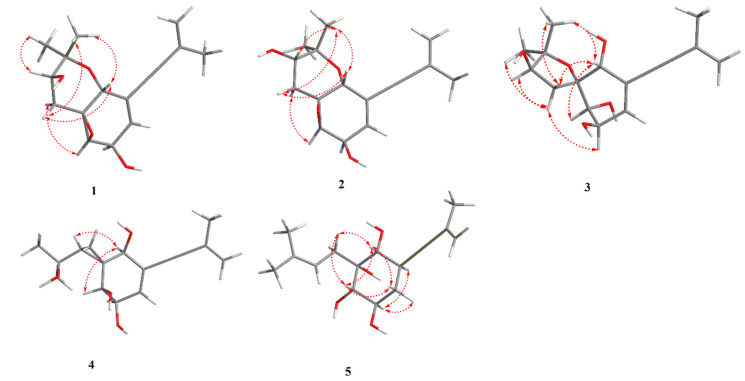
Key NOESY correlations for **1**−**5**.

**Figure 4:**
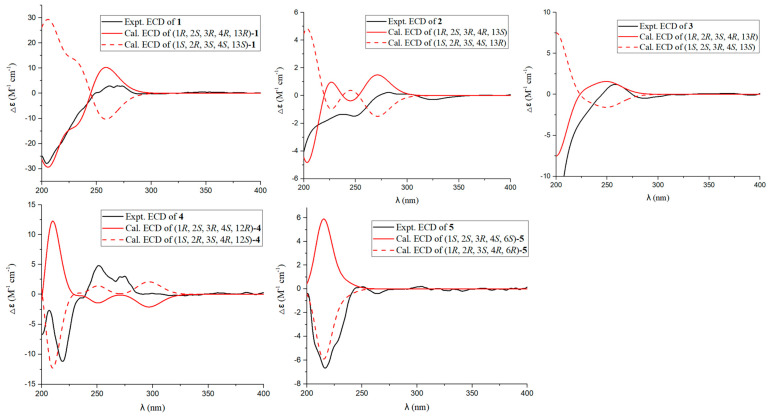
Calculated and experimental ECD spectra of **1**−**5**.

The molecular formula of aspergienyne J (**1**) was defined as C_16_H_20_O_4_ with (+)-HR-ESI-MS ion at *m/z* 299.1247 [M + Na]^+^ (calcd for C_16_H_20_O_4_Na^+^, 299.1254) and ^13^C NMR data, suggesting seven degrees of unsaturation. Combination analysis of ^1^H NMR and HSQC spectra (Table 1) displayed resonances for three olefinic protons [*δ*_H_ 5.93 (1H, dt, *J* = 5.3, 2.0 Hz) and 5.26 (2H, m)], a methylene [*δ*_H_ 2.27 (1H, dd, *J* = 13.3, 12.0 Hz) and 1.57 (1H, dd, *J* = 13.3, 5.0 Hz)], four methines [*δ*_H_ 4.46 (1H, t, *J* = 2.0 Hz), 4.41 (1H, dt, *J* = 5.3, 1.8 Hz), 3.70 (1H, dd, *J* = 12.0, 5.0 Hz) and 3.26 (1H, t, *J* = 1.8 Hz)], and three methyls [*δ*_H_ 1.89 (3H, m), 1.33 (3H, s) and 1.30 (3H, s)]. The ^13^C NMR data (Table 2) showed sixteen carbon resonances, including one sp^2^ methylene carbon (*δ*_C_ 122.3), one sp^2^ methine carbon (*δ*_C_ 133.2), two sp^2^ non-protonated carbons (*δ*_C_ 128.3 and 123.3), two non-protonated sp carbons (*δ*_C_ 93.3 and 86.8), two oxygenated quaternary sp^3^ carbons (*δ*_C_ 78.6 and 61.0), one sp^3^ methylene carbon (*δ*_C_ 36.3), four oxygenated sp^3^ methine carbons (*δ*_C_ 73.2, 67.8, 64.7 and 60.1), and three sp^3^ methyls (*δ*_C_ 28.2, 23.5 and 16.5). Comparison of the NMR data of 1 with those of biscognienyne D (**6**) [5] revealed the same planar structure; however, some proton chemical shifts and coupling constants were different. Furthermore, the ^1^H-^1^H COSY of H-3/H-4 /H-5 and H-12/H-13, as well as the key HMBC signals from H-1 to C-2/C-5/C-6/C-7/C-14, from H-16 to C-1/C-14, were used to further prove the planar structure of 1 (Figure 2). The relative configuration of 1 was established via NOESY experiments. NOESY correlations H-1/H-12a, H-1/H-15, H-12a/H-15, H-13/H-16, and H-3/H-12b (Figure 3), as well as the small *J*_H-3/H-4_ value (1.8 Hz), determined the relative configurations as 1R*, 2S*, 3R*, 4R*, and 13R*. Finally, the comparison of the calculated and experimental ECD spectra allowed us to establish the absolute configurations as 1*R*, 2*S*, 3*R*, 4*R*, and 13*R* (Figure 4).

Aspergienyne K (**2**) has the same molecular formula as **1**, C_16_H_20_O_4_, as determined via (+)-HR-ESI-MS and ^13^C NMR data (Table 2). ^1^H and ^13^C NMR spectra (Table 1 and Table 2) indicated that compound **2** differs to **1** except for a perturbation in the region around carbon at position 13 suggesting a different configuration of the hydroxyl group. The comparison of calculated and experimental ECD spectra confirmed the 13S absolute configuration (Figure 4). 

Aspergienyne L (**3**) was obtained as a yellowish oil, and the molecular formula was assigned as C_16_H_22_O_5_ via (+)-HR-ESI-MS at *m*/*z* 317.1355 [M + Na]^+^ (calcd for C_16_H_22_O_5_Na^+^, 317.1359) and ^13^C NMR data, corresponding to six degrees of unsaturation. The ^13^C NMR and HSQC data of **3** revealed sixteen carbon resonances (Table 2), including two sp carbons, four olefinic carbons, three methyls, one methylene, four methines, and two quaternary carbons. The NMR spectroscopic data of **3** were similar to that of **1**, except that the C-14 was connected to the C-2 through an oxygen atom in **3**. The deduction was corroborated by the key HMBC correlations from H-12/H-13 to C-2/C-14, which demonstrated the existence of a C-2-O-C-14 ring (tetrahydrofuran). NOESY correlations (Figure 3) between H-1 and H-3, between H-1 and H-12b, and between H-12b and H-16 indicated that H-1, H-3, 12b, and H-16 were on the same face. The large J_H-3/H-4_ value (8.1 Hz) and NOESY correlations from H-4 to H-12a/H-13/H-15 suggested the same axial orientation of these protons. Therefore, the relative configuration of **3** was determined as 1R*, 3S*, 4R*, and 13R*. To assign the full relative configuration of **3**, the ^13^C NMR data of two potential isomers (**3a** and **3d**) (Figure S46) were calculated based on the GIAO method, and the (1R, 2R, 3S, 4R, 13R)-configuration of **3** was suggested due to the high DP4+ probability analysis (100%) (Figure S47) and the better correlation coefficient (R^2^ = 0.9982) between the experimental and calculated ^13^C NMR chemical shifts (Figure S48). The absolute configurations of C-1, C-2, C-3, C-4, and C-13 in compound **3** were determined as 1*R*, 2*R*, 3*S*, 4*R*, and 13*R* via comparison of its calculated and experimental ECD spectra (Figure 4).

Aspergienyne M (**4**) was obtained as a yellowish oil. The molecule formula C_16_H_22_O_4_ was established via (+)-HR-ESI-MS at *m*/*z* 301.1409 [M + Na]^+^ (calcd 301.1410) and its ^13^C NMR data (Table 2), corresponding to six degrees of unsaturation. Combination analysis of ^1^H NMR and HSQC spectra (Table 1) of **4** showed signals for three olefinic protons [δ_H_ 6.02 (1H, d, *J* = 3.2 Hz) and 5.26 (2H, m)], one methylene [δ_H_ 0.92 (1H, dd, *J* = 9.7, 4.8 Hz) and 0.81 (1H, dd, *J* = 7.5, 4.8 Hz)], four methines [δ_H_ 4.13 (1H, m), 4.01 (1H, d, *J* = 5.2 Hz), 3.73 (1H, s) and 1.21 (1H, m)], and three methyls [δ_H_ 1.90 (3H, m), 1.36 (3H, s) and 1.25 (3H, s)]. The ^13^C NMR and HSQC experiments of **4** exhibited one sp^2^ methine carbon (δ_C_ 136.2), one non-protonated sp^2^ carbon (δ_C_ 127.5), two non-protonated sp carbons (δ_C_ 92.0, 88.4), two sp^3^ non-protonated carbons (δ_C_ 70.1, 30.6), four sp^3^ methine carbons (δ_C_ 74.1, 73.1, 72.4, 34.3), one sp^3^ methylene carbon (δ_C_ 7.9), and three sp^3^ methyl carbons (δ_C_ 31.7, 29.7, 23.6). The data of **4** (Table 1 and Table 2) were highly similar to those of monosporasol B (**7**) [6], except for the absence of a peroxide bridge. This assumption was supported by the oxymethine (δ_H_/δ_C_ 3.73/74.1, C-1), and the non-protonated carbons (δ_C_ 70.1, C-14) were significantly shifted to the upfield region in **4** compared to **7**. Accordingly, the planar structure of **4** was assigned and confirmed via analysis of key HMBC correlations from H-12 to C-2/C-3, from H-13 to C-1, and from H-15/H-16 to C-13/C-14, (Figure 2). In the NOESY experiment, the correlations from H-1 to H-3 and H-1 to H-13, as well as the small value of J_H-3/H-4_ (5.2 Hz), indicated that H-1, H-3, H-4, and H-13 were on the same face (Figure 3). Therefore, the relative configuration of **4** was established as shown in Figure 3. The absolute configuration 1S, 2R, 3S, 4R, and 12S was deduced via the comparison of calculated and experimental ECD spectra (Figure 4).

The (+)-HR-ESI-MS spectrum of Aspergienyne N (**5**), isolated as a yellowish oil, showed a pseudo molecular ion peak at *m*/*z* 303.1567 [M + Na]^+^ corresponding to a molecular formula C_16_H_24_O_4_. Comprehensive analysis of the NMR spectral information of biscognienyne D (**8**) and **5** (Table 1 and Table 2) indicated that they are structural analogues. The comparison of the NMR data of compounds **5** and **8** [4] revealed the lack of D5,6 double bond and the replacement of the oxirane ring in **8** with two hydroxyl group at C-2 and C-3 in compound **5**. This speculation was further corroborated via two oxymethines at δ_C_ 75.3 (C-2) and 76.3 (C-3), along with the ^1^H-^1^H COSY correlations of H-3/H-4/H-5/H-6 and the crucial HMBC signals from H-6 to C-1/C-5/C-7/C-8 (Figure 2). NOESY correlations between H-1/H-3, H-1/H-5b, H-1/H-12 b, and H-3/H-5 b indicated that each is b positioned. The NOESY correlations from H-4 to H-6 suggested that these protons were on the same side. Therefore, the absolute configurations of C-1, C-2, C-3, C-4, and C-6 in compound **5** were defined as 1*R*, 2*R*, 3*S*, 4*R*, and 6*R* on the basis of the calculated ECD spectrum (Figure 4).

Three known compounds were identified as biscognienyne D (**6**) [5], monosporasol B (**7**) [6], and biscognienyne D (**8**) [4] via comparison of their spectroscopic data with those reported in the literature.

Cytotoxicity of **1**–**8** against five human cancer cell lines, including (T24 (bladder transitional cells), HeLa (cervical carcinoma cells), 5-8F (nasopharyngeal carcinoma cells), MCF-7 (breast cancer cells), and A549 (non-small cell lung cancer cells), was evaluated using the MTT assay. Compound **8** showed strong cytotoxicity against HeLa cancer cells with an IC_50_ value of 11.8 μM, while **3** and **5** showed moderate cytotoxicity against the HeLa cancer cell line, with IC_50_ values of 34.7 and 28.4 μM, respectively, Compounds **1**, **2**, **4**, **6**, and **7** exhibited no noticeable inhibitory activity against all tested cancer cell line, with IC_50_ > 50 μM. Etoposide was used as the positive control (IC_50_: 15. 7 μM).

**Figure 5:**
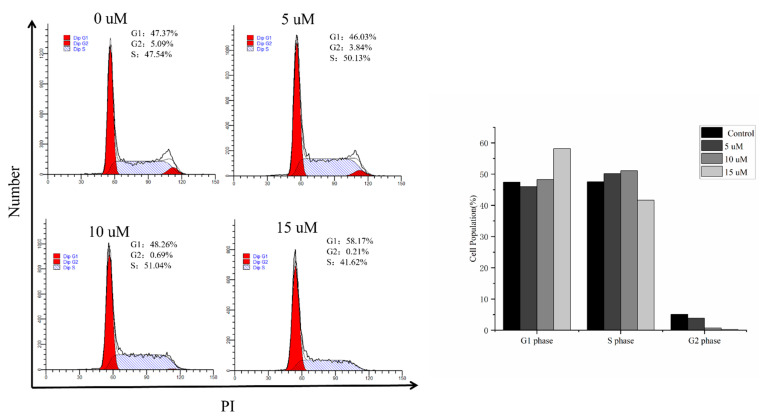
Effects of **8** on the cell cycle distribution in the HeLa cells. The cell cycle distributions of HeLa cells treated with **8** for 24 h were analyzed with flow cytometry. The graphs show the quantified results. Data are presented as the mean fold changes ± SD of three independent experiments (*p* < 0.001).

To verify whether the observed growth inhibition was owing to cell cycle arrest, HeLa cells were treated with **8** (5, 10, and 15 μM) for 24 h, and the distribution of cells in the cell cycle was established based on flow cytometric analysis. As shown in Figure 5, treatment of the HeLa cells with **8** led to an observable increase in the population of cells in the G1 phase (from 46.72% to 57.78%), with a gradual decrease in the cell number in the G2 phase (6.60–0.18%). These findings suggest that **8** may be more likely to induce cell cycle arrest in the G1 phase of HeLa cells.

**Figure 6:**
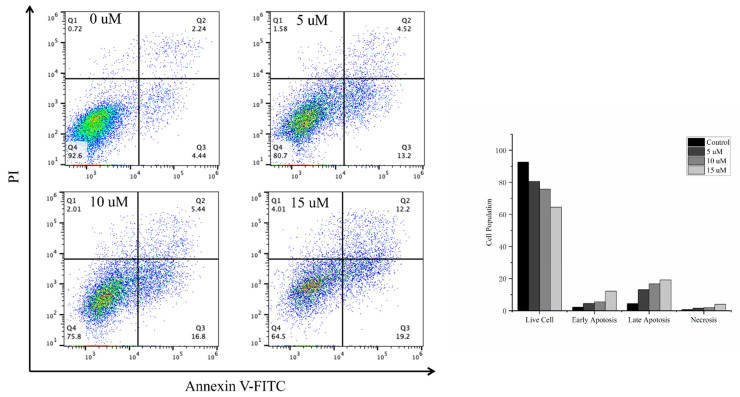
Effects of **8** on the induction of apoptosis in the HeLa cells. Cells were seeded, treated with **8** for 24 h, collected, and stained with annexin V-FITC/PI to detect the apoptotic cell population using flow cytometry. Data are presented as the mean fold changes ± SD of three independent experiments (*p* < 0.001).

To further determine whether the induction of apoptosis contributed to the cytotoxicity in HeLa cells, flow cytometry was performed to detect apoptosis with Annexin V and propidium iodide (PI) double staining. Following treatment of HeLa cells with different concentrations of **8** for 24 h, the percentages of apoptotic cells increased in a concentration-dependent manner to 17.72% (5 μM), 22.24% (10 μM), and 31.4% (15 μM) compared with 6.68% in the control (Figure 6).

These results indicated that the cytotoxicity of **8** against HeLa cells resulted from the cell cycle arrest and the induction of apoptosis.

## 3. Materials and Methods

### 3.1. General Experimental Procedures

The UV and IR spectra of the new compounds were obtained using a PerkinElmer 650 spectrophotometer (PerkinElmer, Waltham, MA, USA) and a PerkinElmer Spectrum Two FT-IR spectrometer (PerkinElmer). The NMR data were acquired on a Bruker 400 MHz (Bruker, Bremen, Germany). Optical rotations were measured using a JASCO P-2000 a polarimeter (Jasco, Tokyo, Japan). An LC-MS spectrometer (Agilent 6545 Q-TOF, Santa Clara, CA, USA) was used to obtain HR-ESI-MS data. The detailed ECD instrument are described in the Supplementary Materials. The other measuring instruments used for the purification of the isolates and the materials used in the separation procedure were identical to our previous reports [9,10].

### 3.2. Fungal Material

The fungal strain GXNU-Y85 was obtained from the fresh fruit of the mangrove plant *Kandelia candel*, in Beihai, and was identified as *Aspergillus* sp. via the sequence of its internal transcribed spacer region (ITS) and morphology. ITSrDNA of GXNU-Y85 was submitted to GenBank, and the accession number is OR999402.

### 3.3. Fermentation, Extraction, and Isolation

The fungal strain GXNU-Y85 was cultured in 80 × 1 L conical flasks, the culture medium mainly consisting of 80 g of rice and 80 mL of water, and the water was 0.5% sea salt. After fermentation for 28 days at a constant temperature of 25 °C, the mycelia were collected and extracted with MeOH (3 × 10 L) to obtain crude extract (21.4 kg), which was then extracted with EtOAc three times. The EtOAc extract was applied on a silica gel VLC column, with the petroleum ether (PE)/EtOAc mixture as eluent (50:1, 30:1, 15:1, 10:1, 5:1, 2:1, 1:1, *v*/*v*), to provide six fractions (Fr. 1–Fr. 6). Fr. 4 (1.34 g) was applied on a Sephadex LH-20 column and eluted with MeOH to obtain six subfractions (Fr. 4.1 to Fr. 4.6). Compounds **5** (4.8 mg), **2** (4.1 mg), and **4** (4.6 mg) were obtained from Fr. 4.3 (0.21 g) through semipreparative HPLC (55% MeOH/H_2_O; 7 mL/min). Purification of Fr. 4.5 (0.31 g) through semipreparative HPLC using MeCN-H_2_O (*v*/*v*, 40:60) as eluent (7 mL/min) yielded **1** (3.6 mg), **3** (3.1 mg), **7** (8.1 mg), **8** (6.9 mg), and **6** (5.4 mg). 

#### 3.3.1. Aspergienyne J (**1**)

Yellowish oil; [α]$\begin{array}{c} 22\\ D \end{array}$ +14.77 (*c* 2.30, MeOH); IR (film) ν_max_ 3400, 2955, 2934, 2871, 2195, 1669, 1457, 1382, 1370, 1055, 1039, 968, 835 cm^−1^; UV (CH_3_OH) λ_max_ (log ε) 258.5 (3.45) nm; ^1^H (400 MHz) and ^13^C (100 MHz) NMR data (in CD_3_OD) (Table 1 and Table 2); HR-ESI-MS *m*/*z* [M + Na]^+^ (calcd for C_16_H_20_O_4_Na^+^ 299.1254; found 299.1247). 

#### 3.3.2. Aspergienyne K (**2**)

Yellowish oil; [α]$\begin{array}{c} 22\\ D \end{array}$ +9.01 (*c* 0.78, MeOH); IR (film) ν_max_ 3426, 2955, 2870, 1712, 1459, 1382, 1370, 1159, 1055, 968, 834, 802 cm^−1^; UV (CH_3_OH) λ_max_ (log ε) 259.4 (3.34) nm; ^1^H (400 MHz) and ^13^C (100 MHz) NMR data (in CD_3_OD) (Table 1 and Table 2); HR-ESI-MS *m*/*z* [M + Na]^+^ (calcd for C_16_H_20_O_4_Na^+^ 299.1254; found 299.1248). 

#### 3.3.3. Aspergienyne L (**3**)

Yellowish oil; [α]$\begin{array}{c} 22\\ D \end{array}$ −6.45 (*c* 1.46, MeOH); IR (film) ν_max_ 3427, 2954, 2870, 1655, 1459, 1382, 1370, 1055, 1038, 968, 834 cm^−1^; UV (CH_3_OH) λ_max_ (log ε) 258.6 (3.58) nm; ^1^H (400 MHz) and ^13^C (100 MHz) NMR data (in CD_3_OD) ( Table 1 and Table 2); HR-ESI-MS *m*/*z* [M + Na]^+^ (calcd for C_16_H_22_O_5_Na^+^ 317.1359; found 317.1355). 

#### 3.3.4. Aspergienyne M (**4**)

Yellowish oil; [α]$\begin{array}{c} 22\\ D \end{array}$ −37.33 (*c* 2.35 MeOH); IR (film) ν_max_ 3408, 2955, 2933, 2871, 2195, 1668, 1457, 1382, 1371, 1256, 1039, 968 cm^−1^; UV (CH_3_OH) λ_max_ (log ε) 258.1 (3.41) nm; ^1^H (400 MHz) and ^13^C (100 MHz) NMR data (in CD_3_OD) (Table 1 and Table 2); HR-ESI-MS *m*/*z* [M + Na]^+^ (calcd for C_16_H_22_O_4_Na^+^ 301.1410; found 301.1409). 

#### 3.3.5. Aspergienyne N (**5**)

Yellowish oil; [α]$\begin{array}{c} 22\\ D \end{array}$ −21.69 (*c* 0.27, MeOH); IR (film) ν_max_ 3417, 2955, 2934, 2871, 2195, 1714, 1669, 1458, 1382, 1370, 1054, 1039, 968, 834 cm^−1^; UV (CH_3_OH) λ_max_ (log ε) 259.2 (3.29) nm; ^1^H (400 MHz) and ^13^C (100 MHz) NMR data (in CD_3_OD) (Table 1 and Table 2); HR-ESI-MS *m*/*z* [M + Na]^+^ (calcd for C_16_H_24_O_4_Na^+^ 303.1567; found 303.1567). 

### 3.4. ECD and NMR Calculations

The ECD and NMR calculations of all the new isolates were performed as previously described [10,11]. The detailed procedure is described in the Supplementary Materials.

### 3.5. Cell Culture

HeLa, T24, 5-8F, MCF-7, and A549 cells were acquired from China Academy of Sciences and were cultured in DMEM medium or RPMI Medium 1640 (RPMI1640) containing 10% (*v*/*v*) fetal bovine serum (FBS) and 1% (*v*/*v*) penicillin sodium salt/streptomycin sulfate. All cells were sustained in a humidified atmosphere at 37 °C with 5% CO_2_. 

### 3.6. Cell Viability Assay

The cytotoxic activity of **1**–**8** against five cancer cell lines were tested via the MTT method as in previous reports [12]. Etoposide was used as the positive control. 

### 3.7. Cell Cycle Analysis

After treatment with **8** for 24 h, HeLa cells were collected, washed with PBS, and fixed with ice-cold 70% EtOH. Propidium iodide (PI) mixed with RNase was used to stain each set of cells, and flow cytometry analysis was carried out as previously reported [12].

### 3.8. Apoptosis Analysis

After 24 h of treatment with **8**, HeLa cells were collected, washed twice with cold PBS, and resuspended in 1 × binding buffer. Annexin V-FITC and PI were used to stain each set of cells, which were then cultivated for 30 min at room temperature. The apoptosis assay was performed based on flow cytometry as described previously [12].

## 4. Conclusions

In summary, aspergienynes J–N (**1**–**5**), five new diisoprenyl cyclohexene-type meroterpenoids, along with three previously described analogues, were isolated from the mangrove endophytic fungus *Aspergillus* sp. GXNU-Y85. Their structures, including the absolute configurations of all the new isolates, were unambiguously established through a combination of HR-ESI-MS and NMR spectroscopic data, as well as ECD calculations. The results showed that the isolates **3**, **5**, and **8** displayed significant cytotoxicity against HeLa cancer cell lines, with IC_50_ values of 11.8–34.7 μM. Furthermore, compound **8** induced apoptosis in HeLa cells. Additionally, compound **8** inhibited the growth and proliferation of HeLa cells and resulted in G1 phase arrest.

## Figures and Tables

**Table 1:** ^1^H NMR (400 MHz) of **1**–**5** in CD_3_OD.

NO	1	2	3	4	5
	*δ*_H_ (*J* in Hz)	*δ*_H_ (*J* in Hz)	*δ*_H_ (*J* in Hz)	*δ*_H_ (*J* in Hz)	*δ*_H_ (*J* in Hz)
1	4.46, t (2.0)	4.42, t (2.4)	4.04, t (2.5)	3.73, s	3.23, d (10.5)
2					
3	3.26, t (1.8)	3.26, t (2.3)	3.51, d (8.1)	4.01, d (5.2)	3.06, d (9.3)
4	4.41, dt (5.3, 1.8)	4.46, dd (4.5, 2.3)	3.97, dt (8.1, 2.8)	4.13, m	3.65, m
5a	5.93, dt (5.3, 2.0)	5.73, dd (4.5, 2.4)	5.85, dd (2.8, 2.5)	6.02, d (3.2)	2.04, dt (13.2, 4.6)
5b	5.93, dt (5.3, 2.0)	5.73, dd (4.5, 2.4)	5.85, dd (2.8, 2.5)	6.02, d (3.2)	1.26, m
6					2.83, m
7					
8					
9					
10	5.26, m	5.24, m	5.28, m	5.26, m	5.19, m
					5.15, m
11	1.89, m	1.88, m	1.90, m	1.90, m	1.86 m
12a	2.27, dd (13.3, 12.0)	2.72, dd (14.8, 3.3)	1.79, dd (13.9, 1.8)	0.92, dd (9.7, 4.8)	2.51, dd (13.4, 7.9)
12b	1.57, dd (13.3, 5.0)	1.55, dd (14.8, 2.7)	2.48, dd (13.9, 6.3)	0.81, dd (7.5, 4.8)	2.42, dd (13.4, 7.8)
13	3.70, dd (12.0, 5.0)	3.62, dd (3.3, 2.7)	3.87, dd (6.3, 1.8)	1.21, m	5.18, m
14					
15	1.30, s	1.32, s	1.26, s	1.36, s	1.74, s
16	1.33, s	1.37, s	1.27, s	1.25, s	1.71, s

**Table 2:** ^13^C NMR (100 MHz) of **1**–**5** in CD_3_OD.

NO	1	2	3	4	5
	*δ*_C_, Type	*δ*_C_, Type	*δ*_C_, Type	*δ*_C_, Type	*δ*_C_, Type
1	67.8, CH	68.4, CH	74.0, CH	74.1, CH	74.7, CH
2	61.0, C	57.4, C	89.8, C	30.6, C	78.4, C
3	60.1, CH	58.6, CH	76.9, CH	73.1, CH	76.2, CH
4	64.7, CH	66.9, CH	73.0, CH	72.4, CH	70.9, CH
5	133.2, CH	134.7, CH	135. 6, CH	136.2, CH	36.9, CH_2_
6	123.3, C	121.6, C	126.9, C	127.5, C	33.0, CH
7	86.8, C	86.7, C	87.1, C	88.4, C	83.5, C
8	93.3, C	92.3, C	92.4, C	92.0, C	91.6, C
9	128.3, C	128.5, C	128.3, C	128.3, C	128.8, C
10	122.3, CH_2_	121.9, CH_2_	122.5, CH_2_	122.3, CH_2_	121.0, CH_2_
11	23.5, CH_3_	23.5, CH_3_	23.5, CH_3_	23.6, CH_3_	24.0, CH_3_
12	36.3, CH_2_	34.9, CH_2_	33.7, CH_2_	7.9, CH_2_	33.8, CH_2_
13	73.2, CH	72.4, CH	78.8, CH	34.3, CH	120.1, CH
14	78.6, C	78.0, C	86.5, C	70.1, C	136.1, C
15	16.5, CH_3_	26.4, CH_3_	28.3, CH_3_	31.7, CH_3_	26.3, CH_3_
16	28.2, CH_3_	22.3, CH_3_	23.8, CH_3_	29.7, CH_3_	18.2, CH_3_

## Data Availability

The authors declare that all data of this study are available within the article and its Supplementary Materials file or from the corresponding authors upon request.

## Supplementary Materials

The following supporting information can be downloaded at: https://www.mdpi.com/article/10.3390/md22020058/s1, NMR and HRESIMS spectra of **1**–**5**.
