# Supplementary material for: Diisoprenyl Cyclohexene-Type Meroterpenoids with Cytotoxic Activity from a Mangrove Endophytic Fungus Aspergillus sp. GXNU-Y85
Source: Mar Drugs. 2024 Jan 24;22(2):58. doi: 10.3390/md22020058 (PMC10890295; doi:10.3390/md22020058)
Supplement: Supplementary file 1 [file marinedrugs-22-00058-s001.zip › marinedrugs-2813790-supplementary.pdf]

Supporting Information

Diisoprenyl Cyclohexene-Type Meroterpenoids with Cytotoxic Activity from a Mangrove Endophytic Fungus Aspergillus sp. GXNU-Y85

Feng Qin 1,2,†, Zi-Shuo Song 1,†, Li Luo 1, Xiang-Long Bo 1, Fu-Rong Wu 1, Mei-Jing Tan 1, Fan-Fan Wang 2, Xi-Shan Huang 1,* and Heng-Shan Wang 1,*

1 State Key Laboratory for Chemistry and Molecular Engineering of Medicinal Resources/Key Laboratory for Chemistry and Molecular Engineering of Medicinal Resources (Ministry of Education of China), Collaborative Innovation Center for Guangxi Ethnic Medicine, School of Chemistry and Pharmaceutical Sciences, Guangxi Normal University, Guilin 541004, China; qinf1114@163.com (F.Q.); zishuos@163.com (Z.-S.S.); luoli51598@163.com (L.L.); bxl4937@foxmail.com (X.-L.B.); xingkong2137@gmail.com (F.-R.W.); tanmeijing2022@163.com (M.-J.T.)

2 Guangxi Key Laboratory of Agricultural Resources Chemistry and Biotechnology, College of Chemistry and Food Science, Yulin Normal University, Yulin 537000, China; fanfanwang2020@163.com

* Correspondence: huangxishan13@foxmail.com (X.-S.H.); whengshan@163.com (H.-S.W.)

† These authors contributed equally to this work.

Contents

Figure S1 ^{1}H NMR spectrum of compound **1** in CD_3OD 4
Figure S2 ^{13}C NMR spectrum of compound **1** in CD_3OD 4
Figure S3 ^{1}H-^{1}H COSY spectrum of compound **1** in CD_3OD 5
Figure S4 HSQC spectrum of compound **1** in CD_3OD 5
Figure S5 HMBC spectrum of compound **1** in CD_3OD 6
Figure S6 NOESY spectrum of compound **1** in CD_3OD 6
Figure S7 HR-ESI-MS spectrum of compound **1** 7
Figure S8 UV spectrum of compound **1** 7
Figure S9 IR spectrum of compound **1** 8
Figure S10 ^{1}H NMR spectrum of compound **2** in CD_3OD 8
Figure S11 ^{13}C NMR spectrum of compound **2** in CD_3OD 9
Figure S12 ^{1}H-^{1}H COSY spectrum of compound **2** in CD_3OD 9
Figure S13 HSQC spectrum of compound **2** in CD_3OD 10
Figure S14 HMBC spectrum of compound **2** in CD_3OD 10
Figure S15 NOESY spectrum of compound **2** in CD_3OD 11
Figure S16 HR-ESI-MS spectrum of compound **2** 11
Figure S17 UV spectrum of compound **2** 12
Figure S18 IR spectrum of compound **2** 12
Figure S19 ^{1}H NMR spectrum of compound **3** in CD_3OD 13
Figure S20 ^{13}C NMR spectrum of compound **3** in CD_3OD 13
Figure S21 ^{1}H-^{1}H COSY spectrum of compound **3** in CD_3OD 14
Figure S22 HSQC spectrum of compound **3** in CD_3OD 14
Figure S23 HMBC spectrum of compound **3** in CD_3OD 15
Figure S24 NOESY spectrum of compound **3** in CD_3OD 15
Figure S25 HR-ESI-MS spectrum of compound **3** 16
Figure S26 UV spectrum of compound **3** 16
Figure S27 IR spectrum of compound **3** 17
Figure S28 ^{1}H NMR spectrum of compound **4** in CD_3OD 17
Figure S29 ^{13}C NMR spectrum of compound **4** in CD_3OD 18
Figure S30 ^{1}H-^{1}H COSY spectrum of compound **4** in CD_3OD 18
Figure S31 HSQC spectrum of compound **4** in CD_3OD 19
Figure S32 HMBC spectrum of compound **4** in CD_3OD 19
Figure S33 NOESY spectrum of compound **4** in CD_3OD 20
Figure S34 HR-ESI-MS spectrum of compound **4** 20
Figure S35 UV spectrum of compound **4** 21
Figure S36 IR spectrum of compound **4** 21
Figure S37 ^{1}H NMR spectrum of compound **5** in CD_3OD 22
Figure S38 ^{13}C NMR spectrum of compound **5** in CD_3OD 22
Figure S39 ^{1}H-^{1}H COSY spectrum of compound **5** in CD_3OD 23
Figure S40 HSQC spectrum of compound **5** in CD_3OD 23
Figure S41 HMBC spectrum of compound **5** in CD_3OD 24
Figure S42 NOESY spectrum of compound **5** in CD_3OD 24
Figure S43 HR-ESI-MS spectrum of compound **5** 25

Figure S44 UV spectrum of compound **5** 25
Figure S45 IR spectrum of compound **5** 26
Computational Section...26
ECD calculation 26
Table S1. Energies of the dominative conformers of compounds **1-5.** 27
^{13}C NMR calculation...28
Figure S46 Conformations of low-energy conformers of structures **3a** and **3b**. 28
Table S2 DP4+ analysis result table of model compounds **3a** and **3b**.........................29
Figure S47 DP4+ probability analysis result. 30
Table S3. ^{1}H (400 MHz) and ^{13}C (100 MHz) NMR, and COSY, HMBC and NOESY assignment of **1** in CD_3OD. 30
Table S4. ^{1}H (400 MHz) and ^{13}C (100 MHz) NMR, and COSY, HMBC and NOESY assignment of **2** in CD_3OD. 31
Table S5. ^{1}H (400 MHz) and ^{13}C (100 MHz) NMR, and COSY, HMBC and NOESY assignment of **3** in CD_3OD. 31
Table S6. ^{1}H (400 MHz) and ^{13}C (100 MHz) NMR, and COSY, HMBC and NOESY assignment of **4** in CD_3OD. 32
Table S7. ^{1}H (400 MHz) and ^{13}C (100 MHz) NMR, and COSY, HMBC and NOESY assignment of **5** in CD_3OD. 32
Table S8. ^{1}H (400 MHz) and ^{13}C (100 MHz) NMR of **6-8** in CD_3OD 32

Figure S1 ^{1}H NMR spectrum of compound **1** in CD_3OD

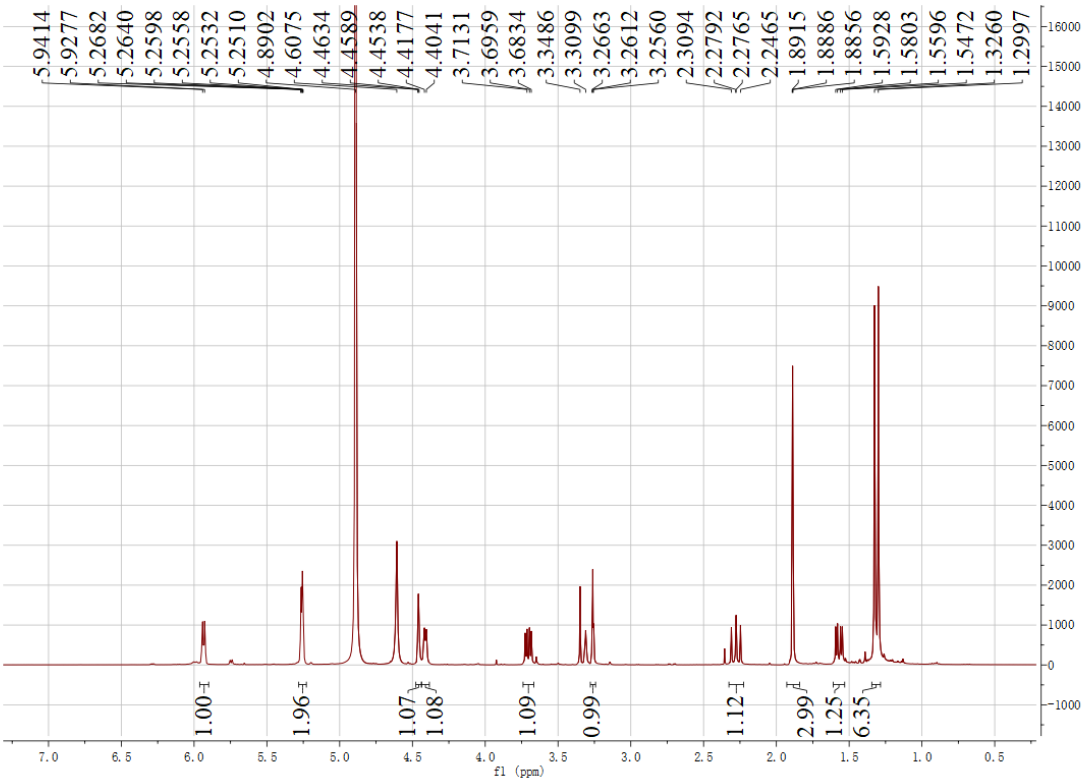

Figure S2 ^{13}C NMR spectrum of compound **1** in CD_3OD

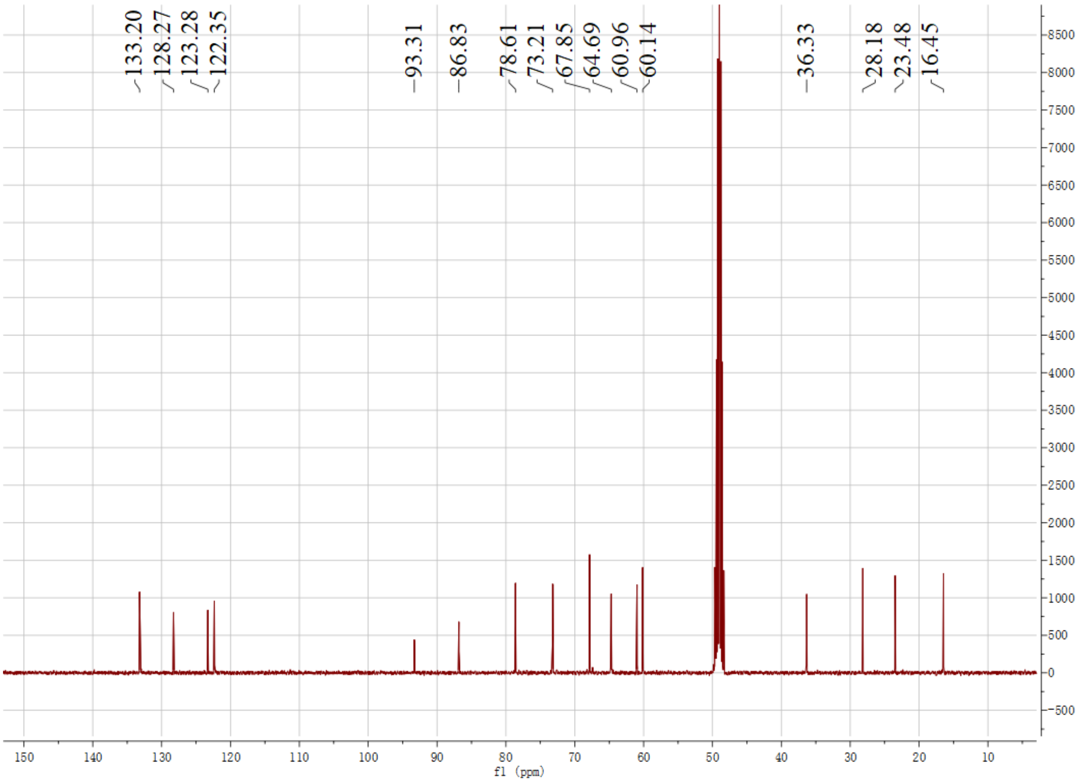

Figure S3 ^{1}H-^{1}H COSY spectrum of compound **1** in CD_3OD

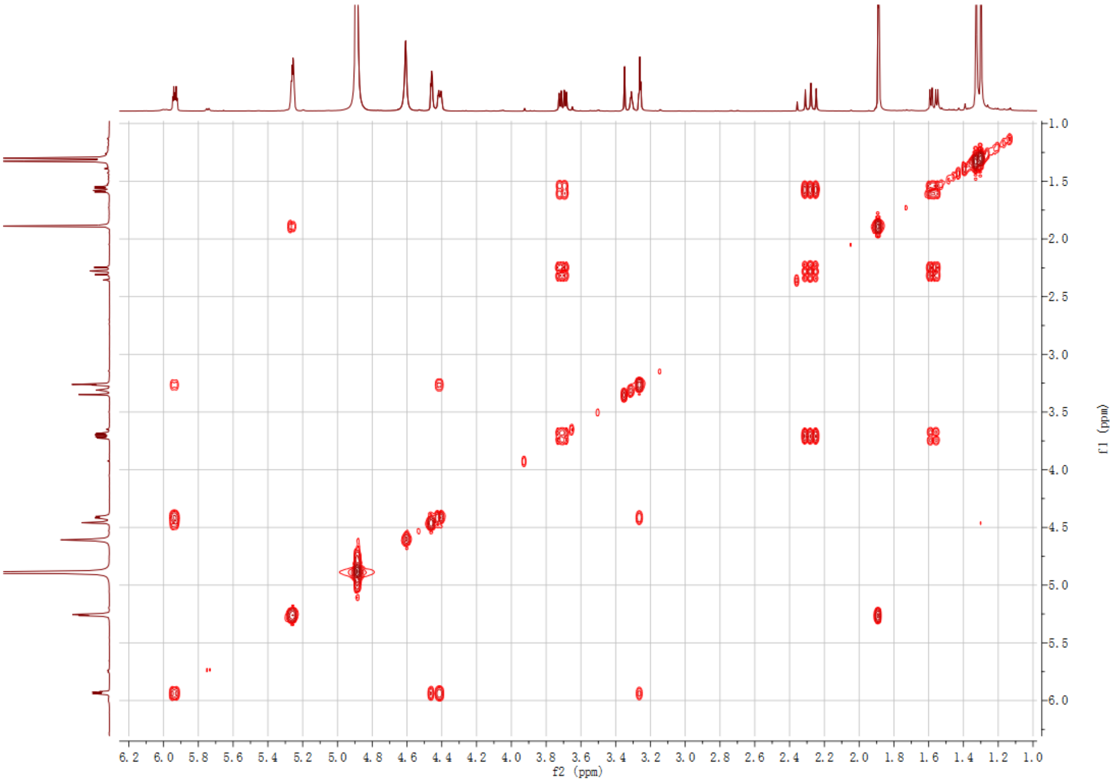

Figure S4 HSQC spectrum of compound **1** in CD_3OD

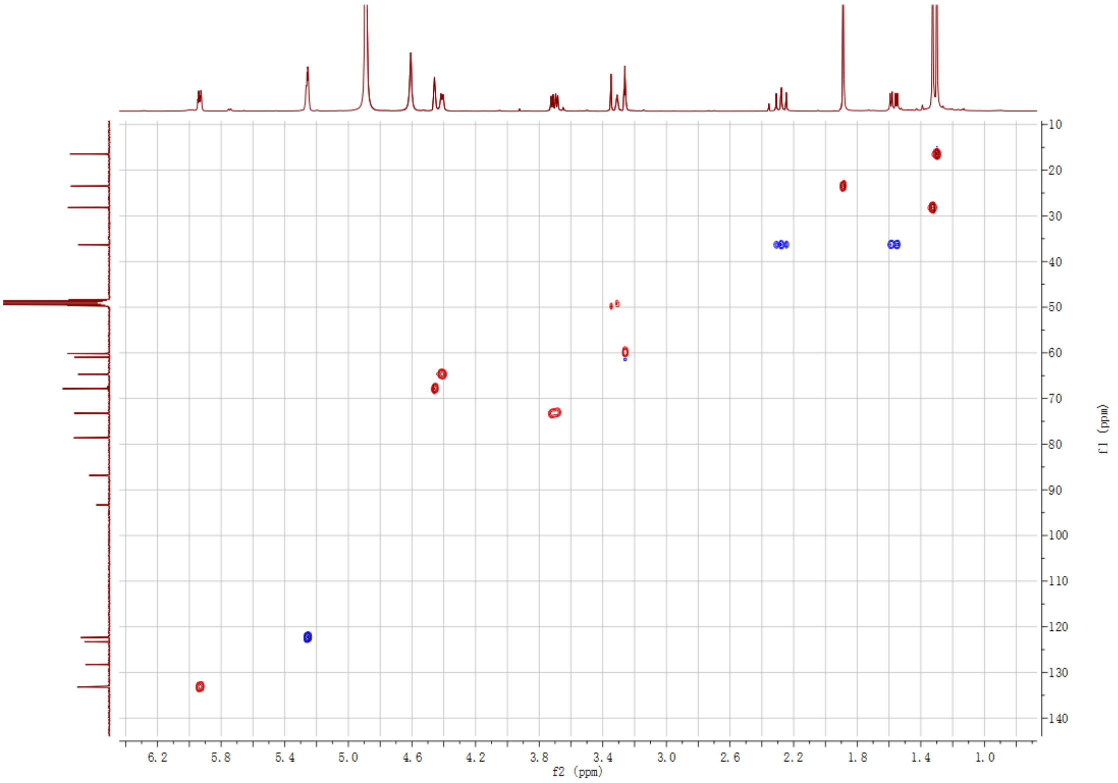

Figure S5 HMBC spectrum of compound **1** in CD_3OD

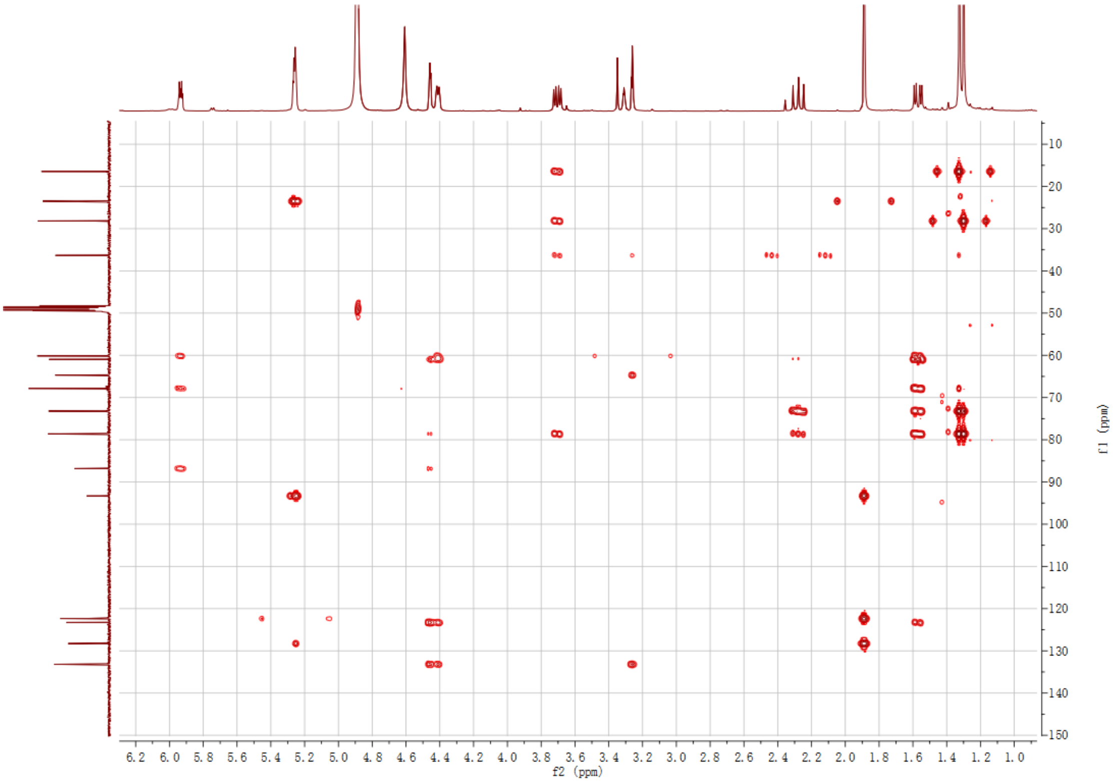

Figure S6 NOESY spectrum of compound **1** in CD_3OD

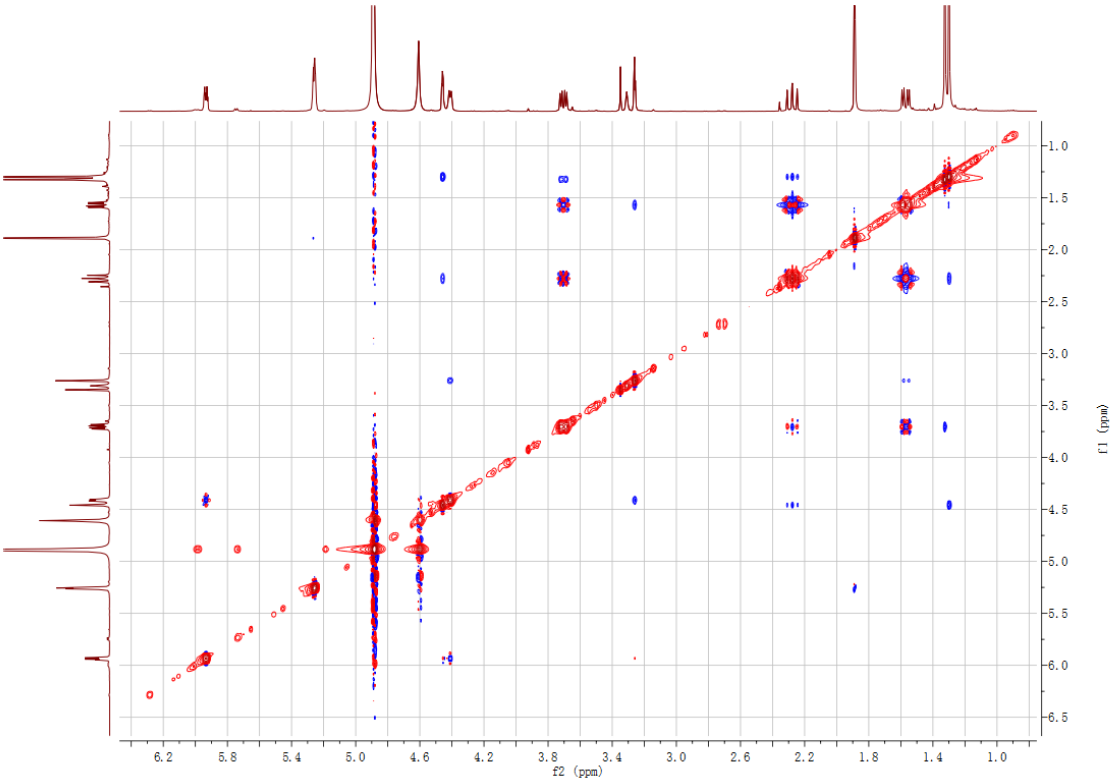

Figure S7 HR-ESI-MS spectrum of compound **1**

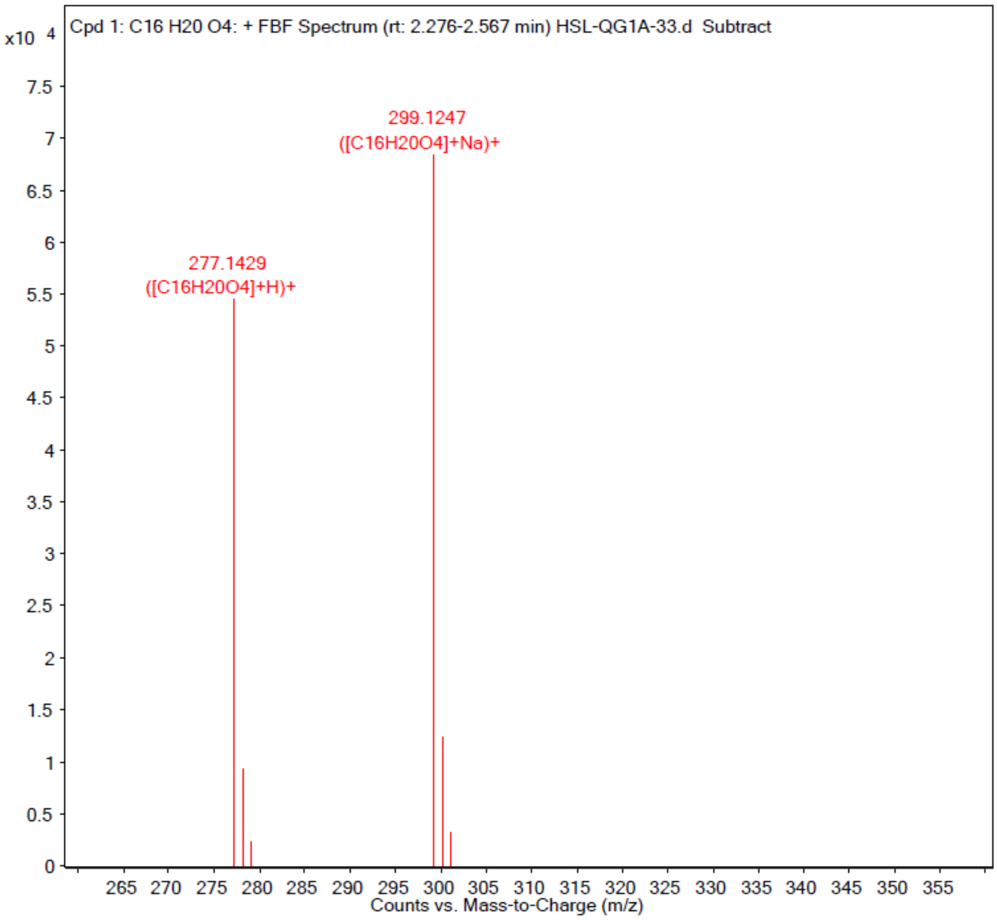

Figure S8 UV spectrum of compound **1**

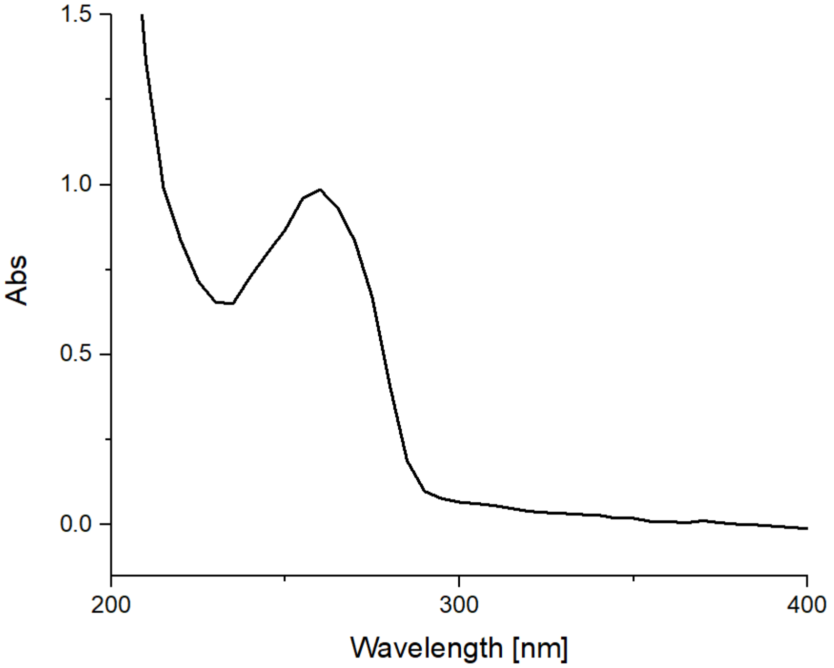

Figure S9 IR spectrum of compound **1**

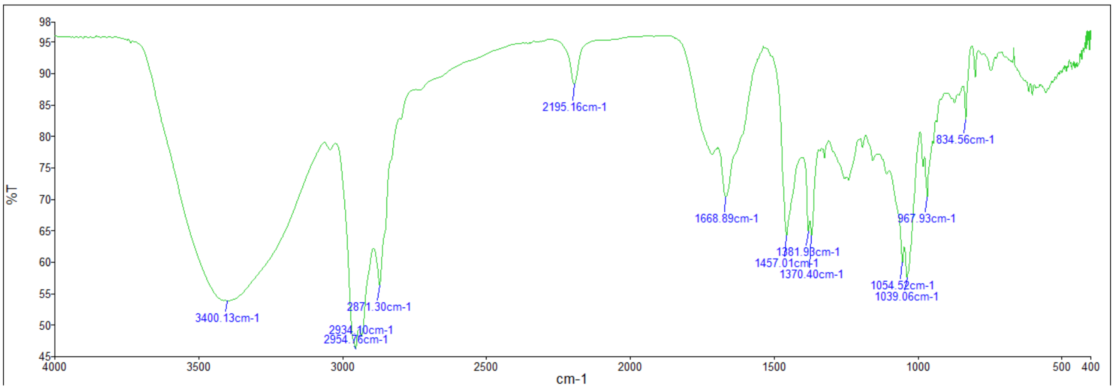

Figure S10 ^{1}H NMR spectrum of compound **2** in CD_3OD

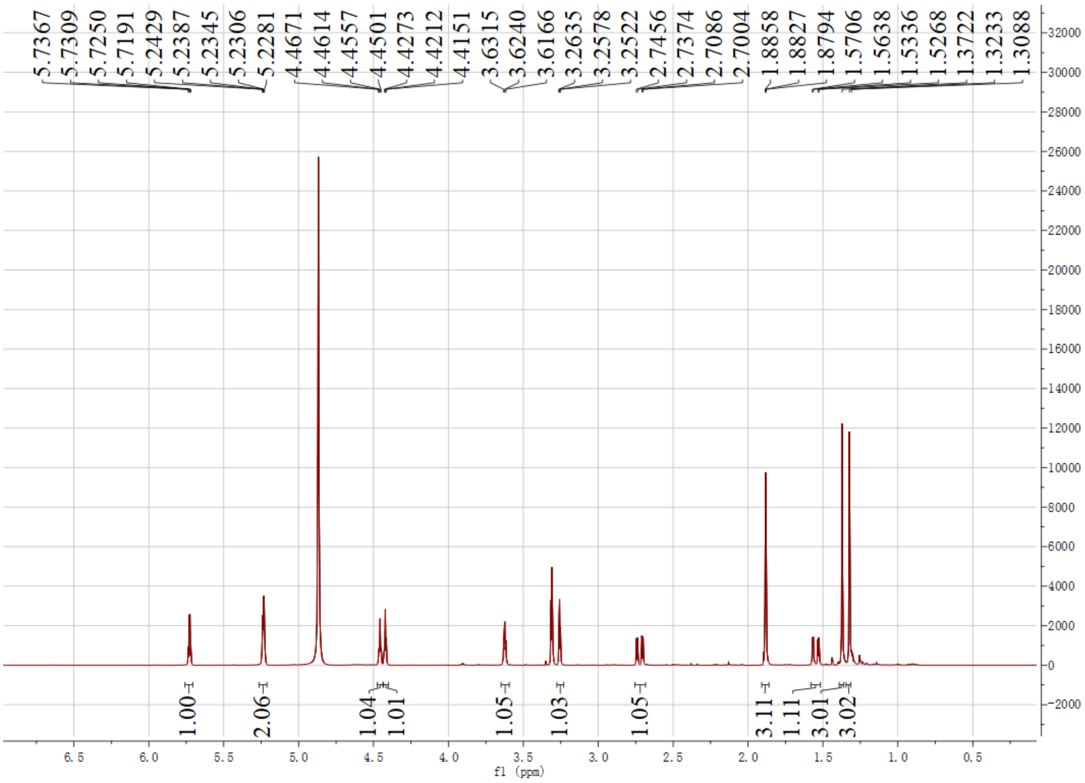

Figure S11 ^{13}C NMR spectrum of compound **2** in CD_3OD

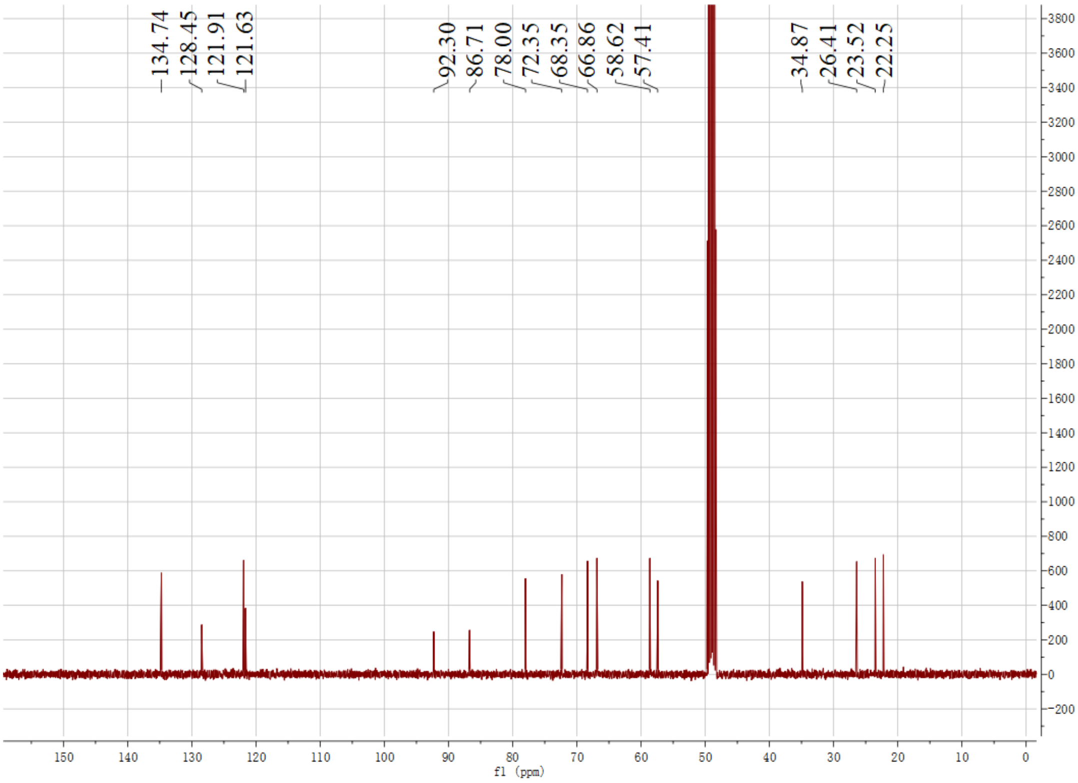

Figure S12 ^1H-^1H COSY spectrum of compound **2** in CD_3OD

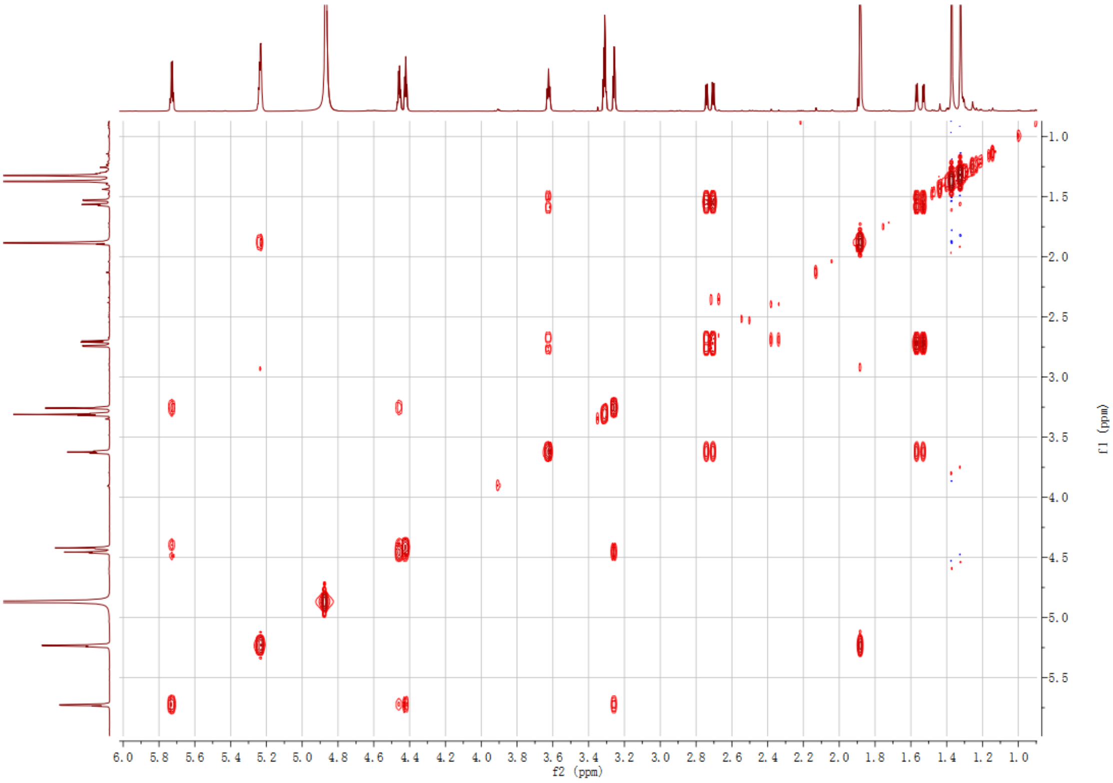

Figure S13 HSQC spectrum of compound **2** in CD_3OD

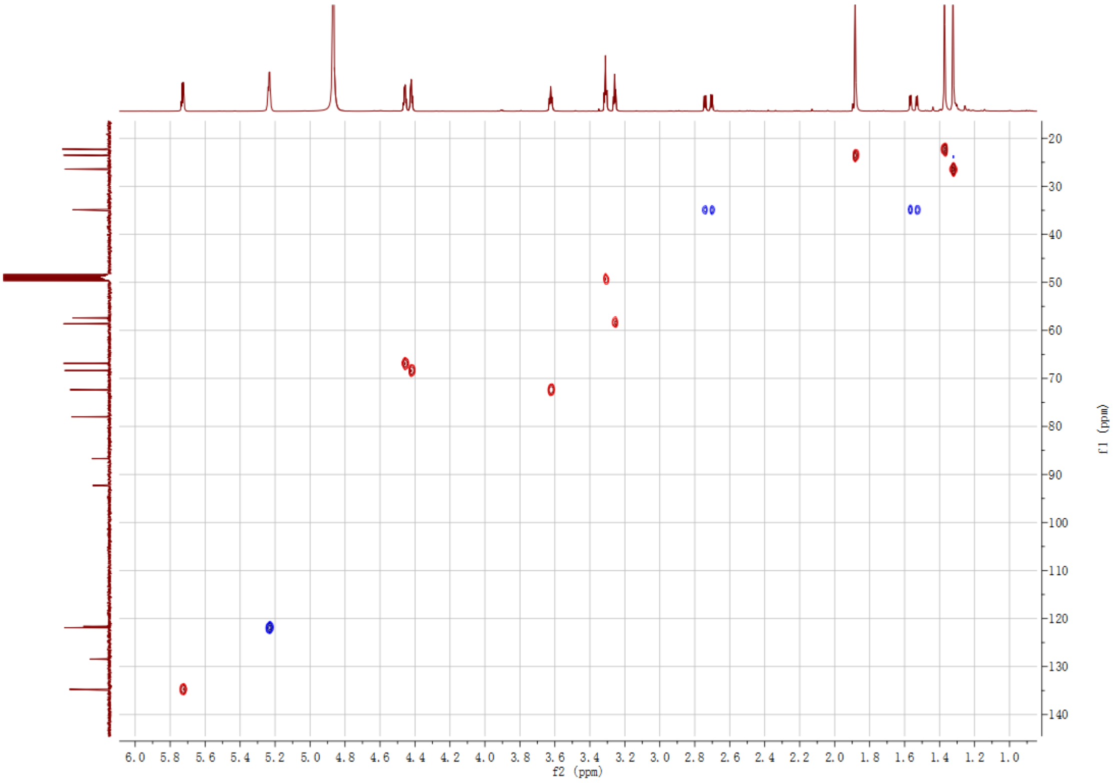

Figure S14 HMBC spectrum of compound **2** in CD_3OD

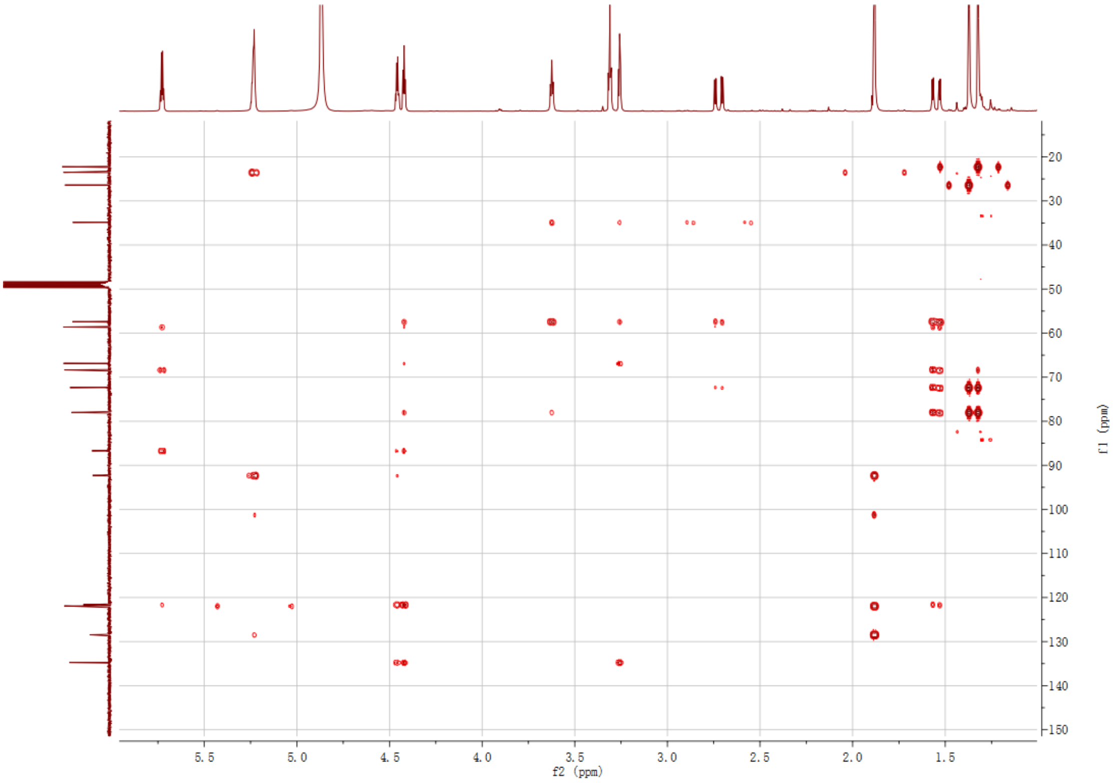

Figure S15 NOESY spectrum of compound **2** in CD_3OD

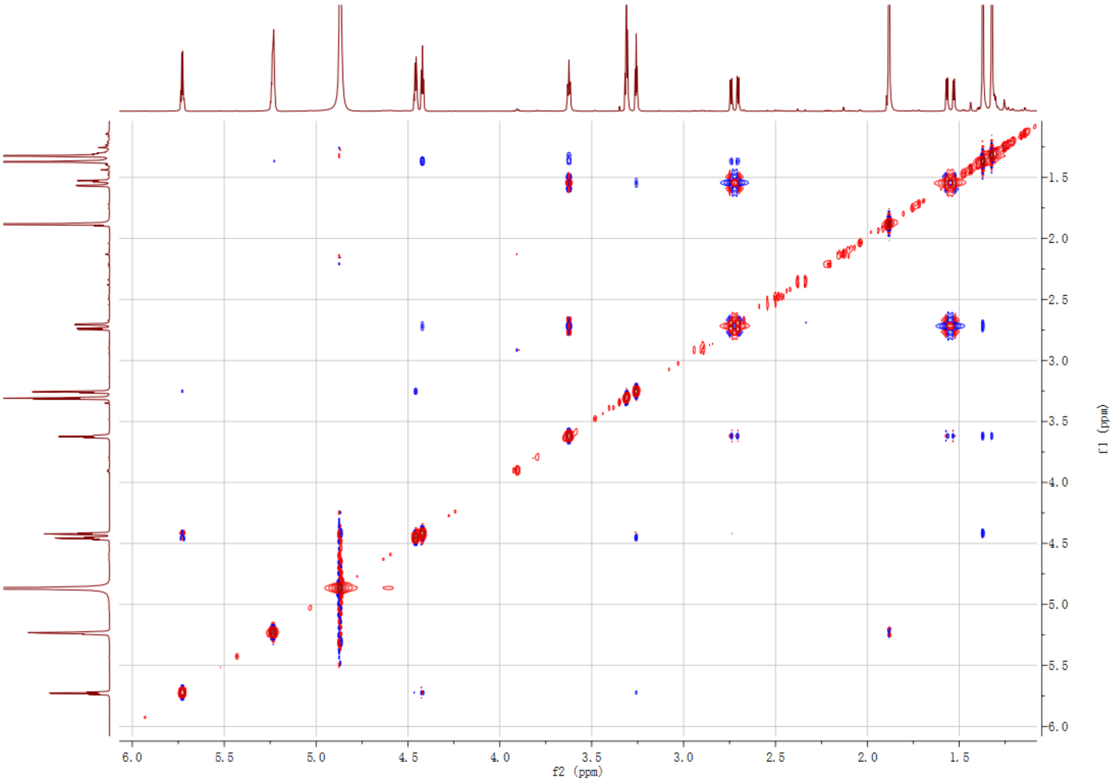

Figure S16 HR-ESI-MS spectrum of compound **2**

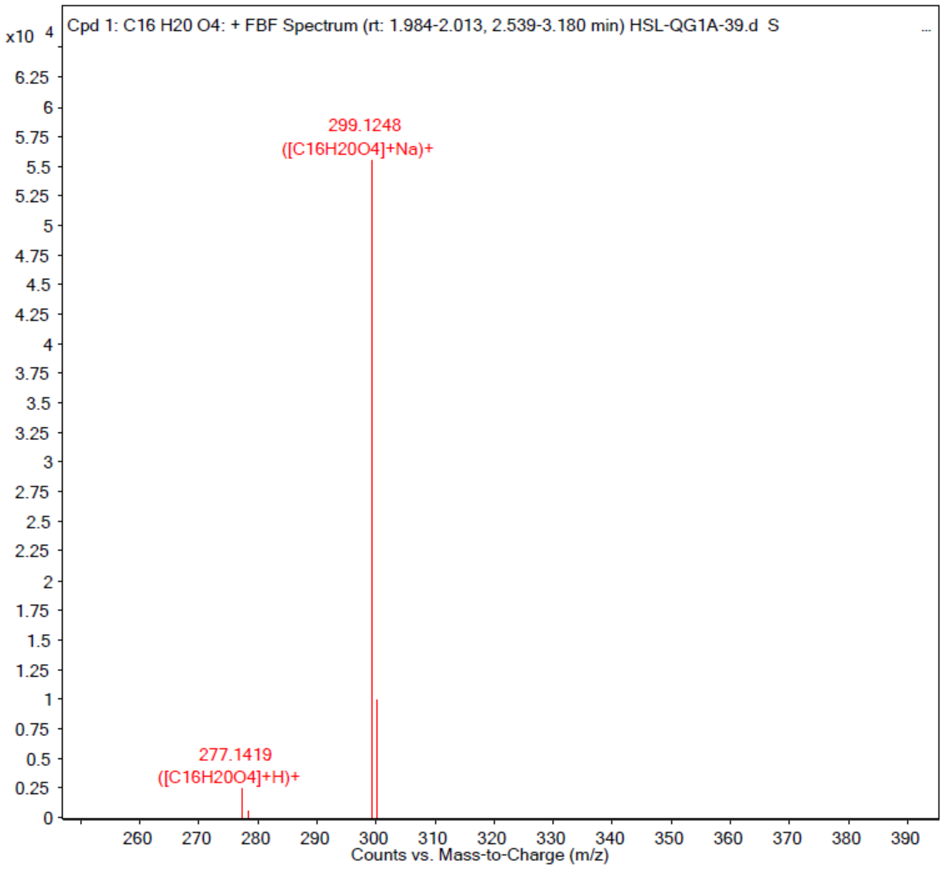

Figure S17 UV spectrum of compound **2**

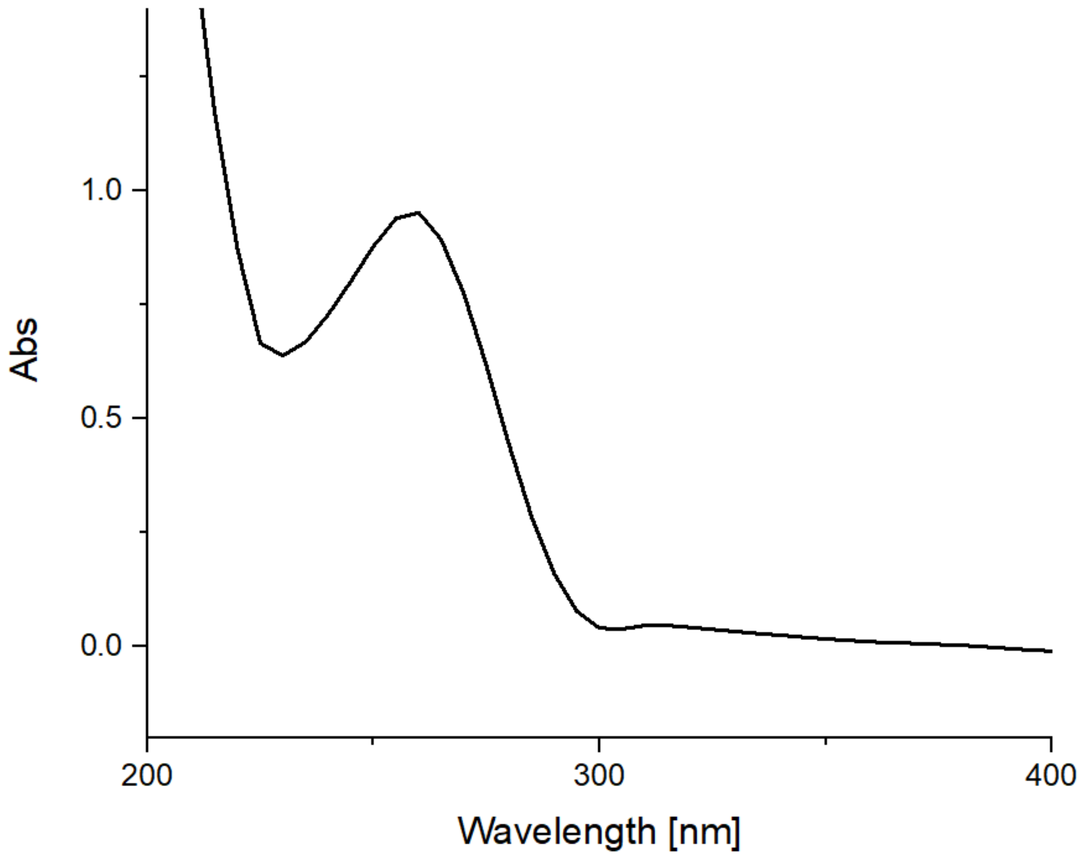

Figure S18 IR spectrum of compound **2**

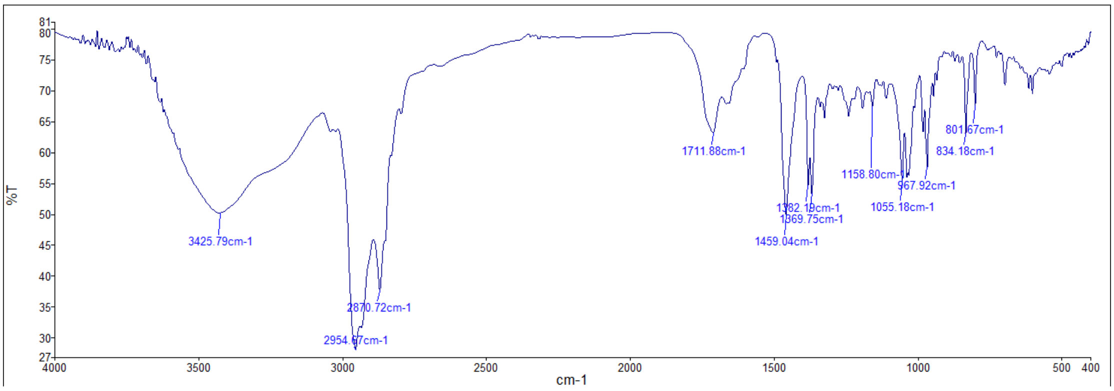

Figure S19 ^{1}H NMR spectrum of compound **3** in CD_3OD

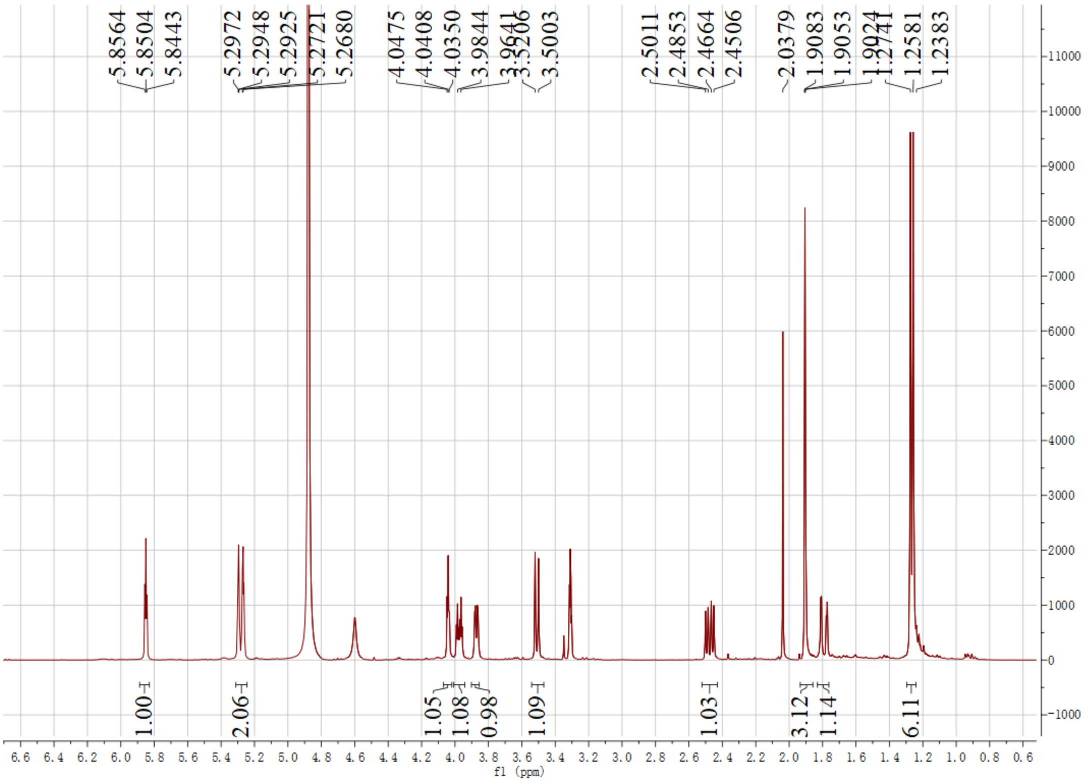

Figure S20 ^{13}C NMR spectrum of compound **3** in CD_3OD

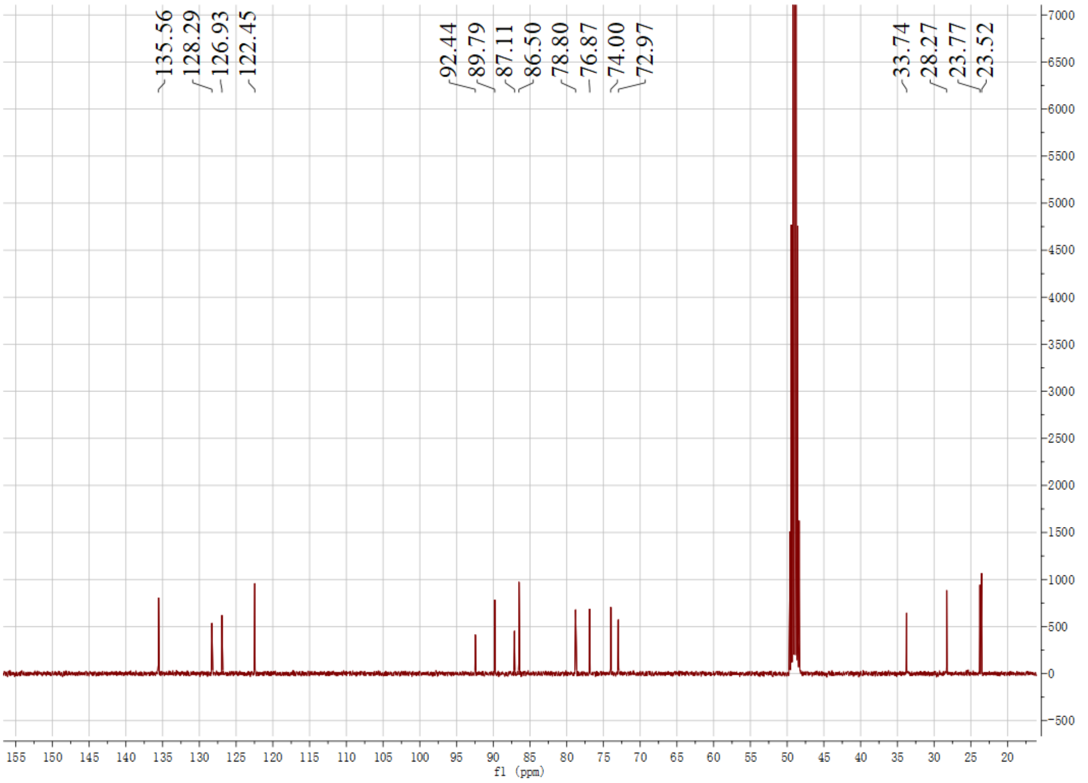

Figure S21 ^{1}H-^{1}H COSY spectrum of compound **3** in CD_3OD

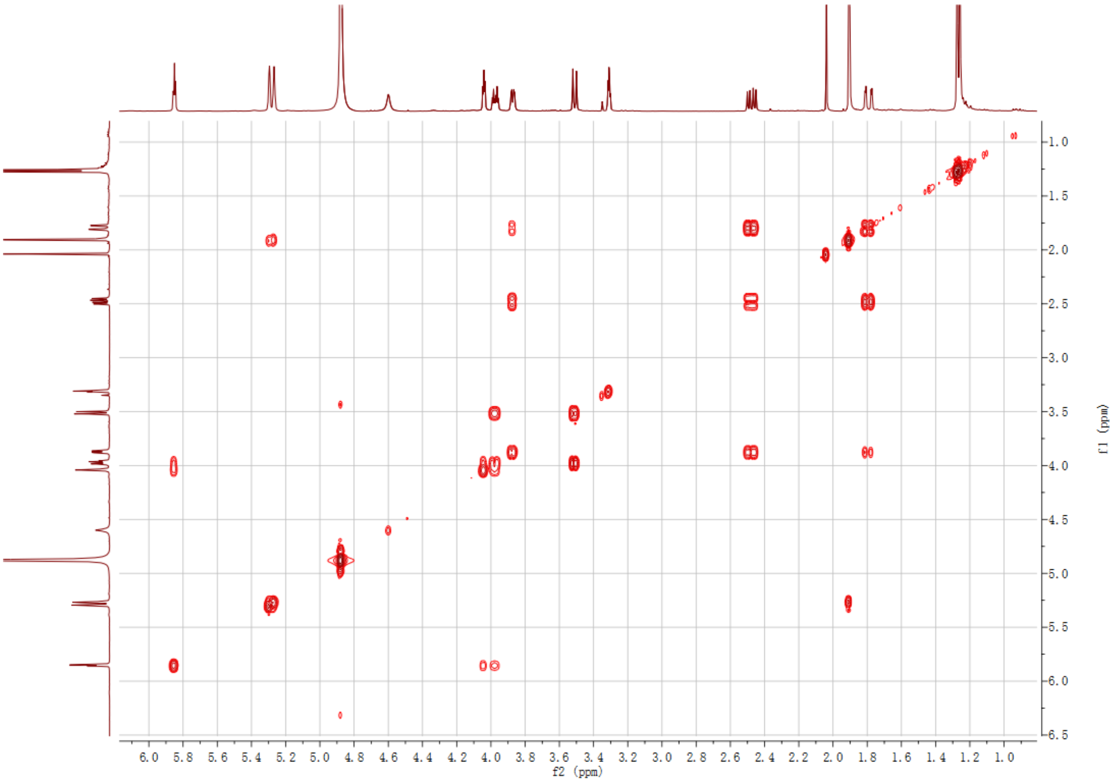

Figure S22 HSQC spectrum of compound **3** in CD_3OD

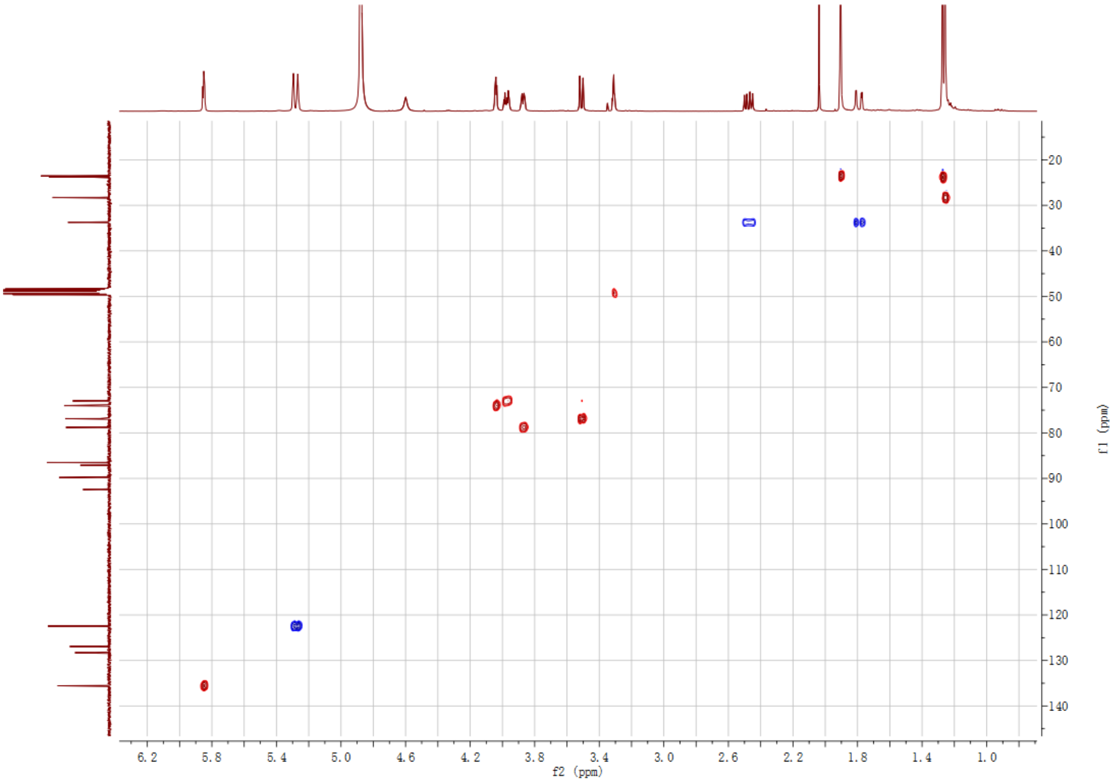

Figure S23 HMBC spectrum of compound **3** in CD_3OD

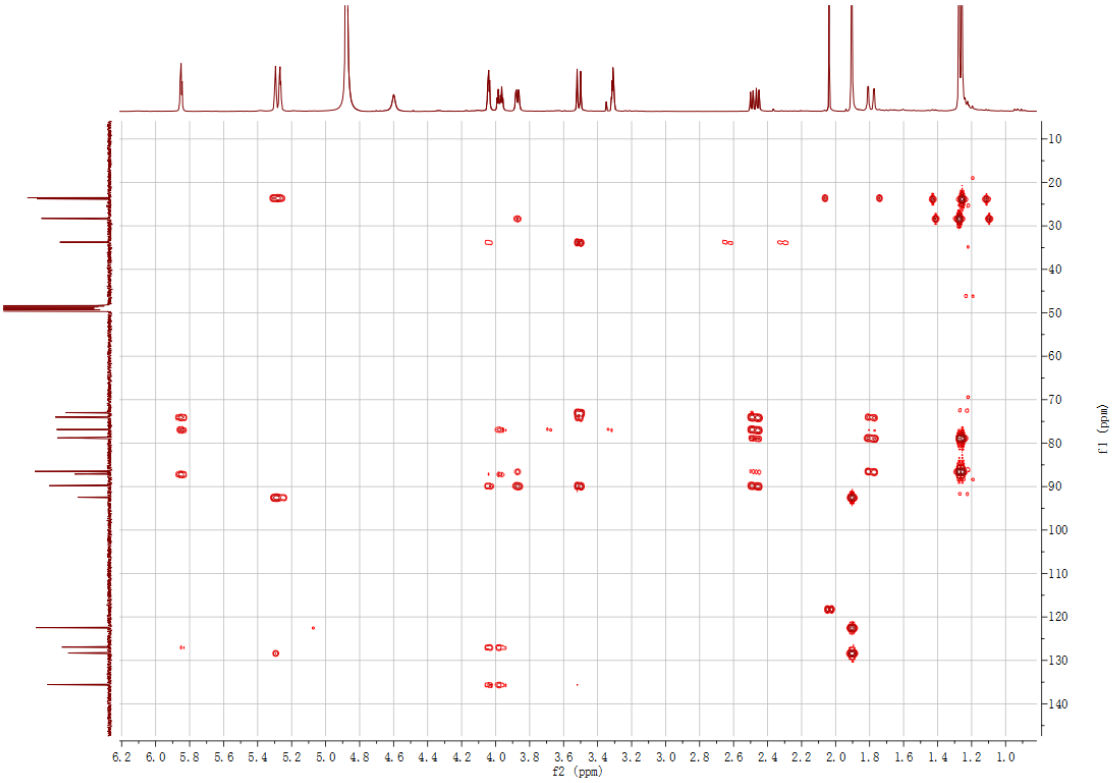

Figure S24 NOESY spectrum of compound **3** in CD_3OD

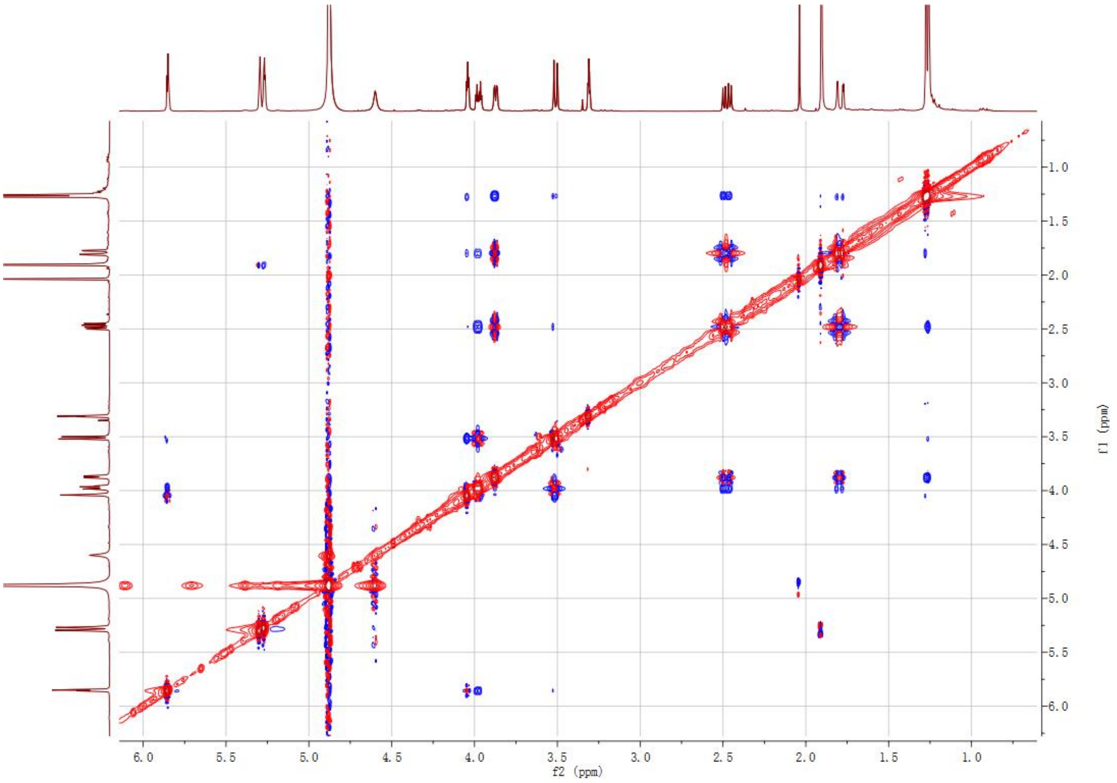

Figure S25 HR-ESI-MS spectrum of compound **3**

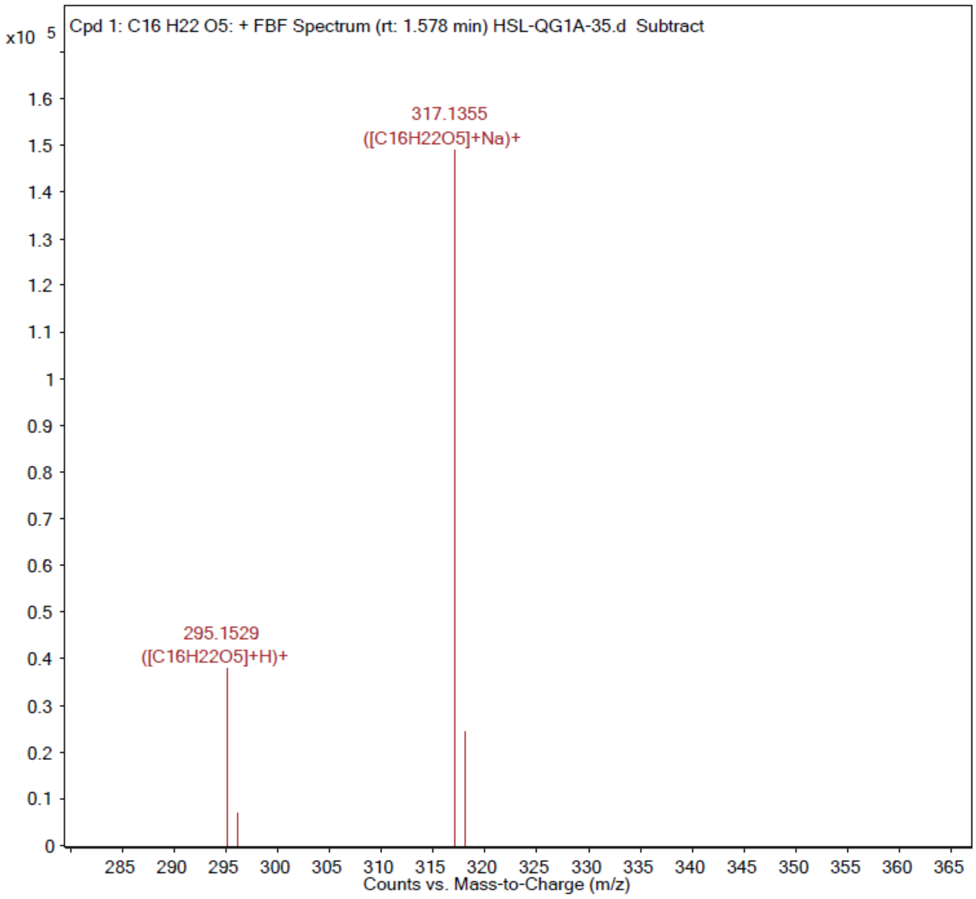

Figure S26 UV spectrum of compound **3**

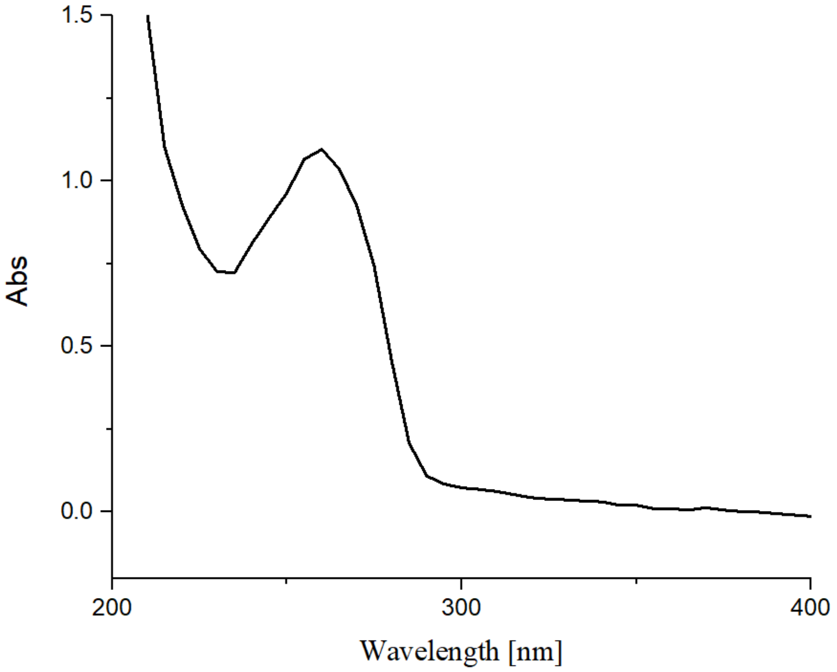

Figure S27 IR spectrum of compound **3**

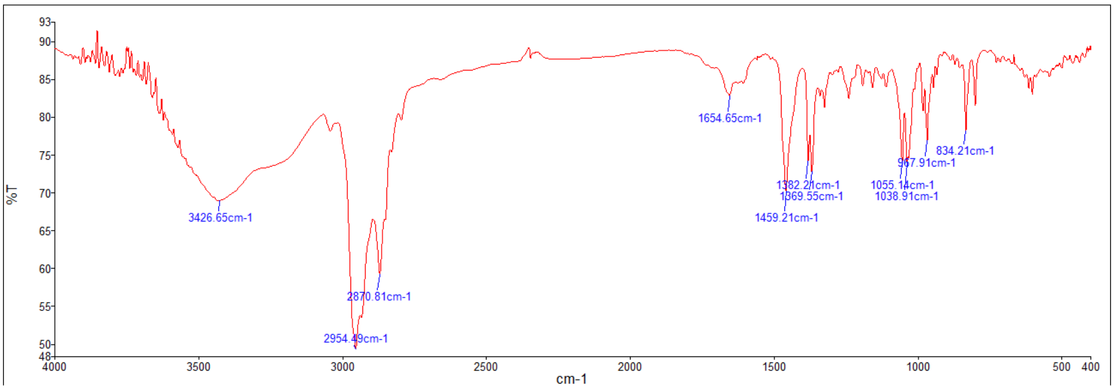

Figure S28 ^{1}H NMR spectrum of compound **4** in CD_3OD

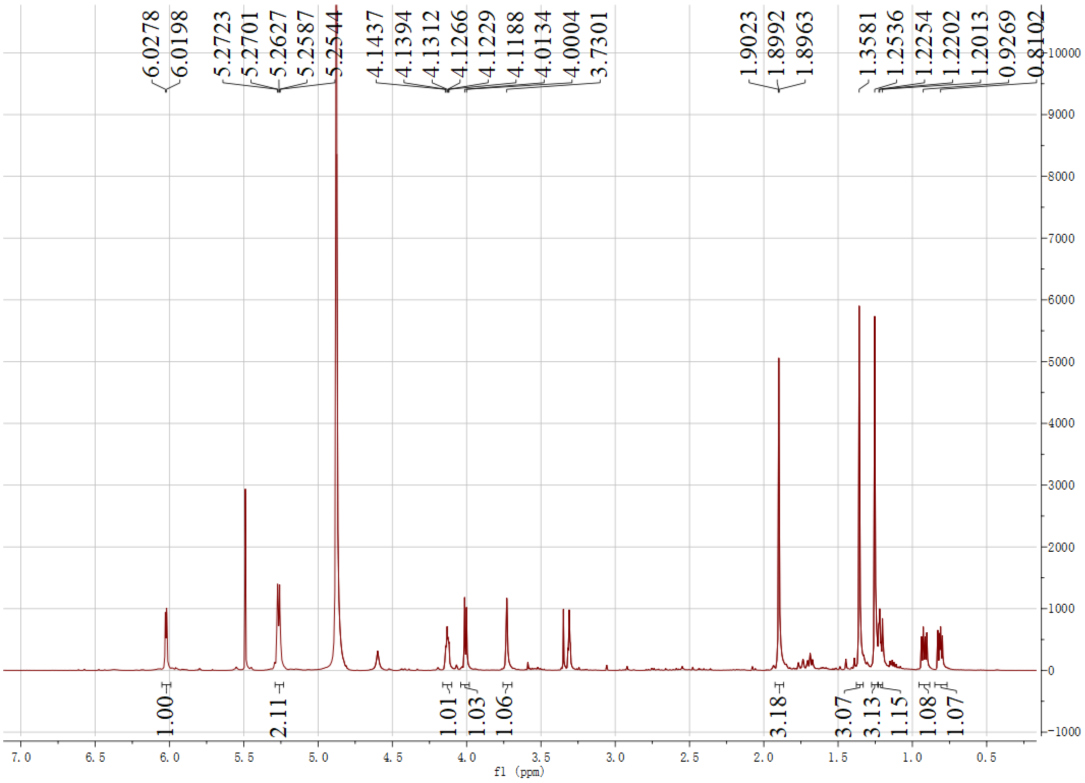

Figure S29 ^{13}C NMR spectrum of compound **4** in CD_3OD

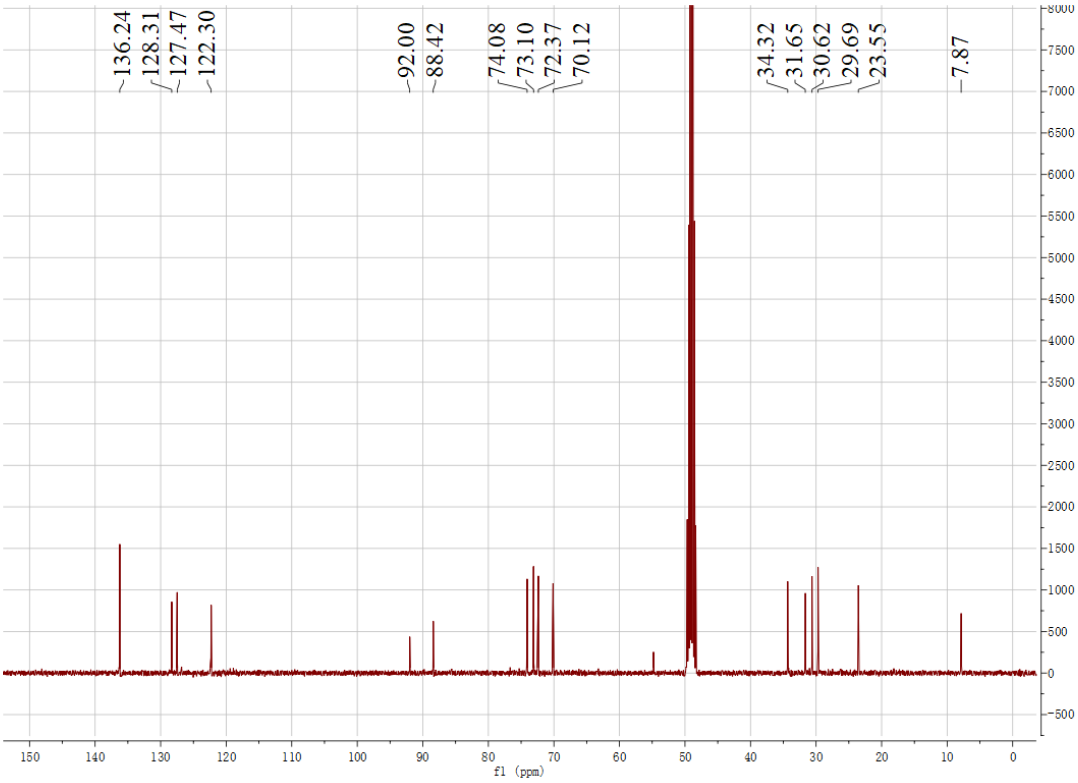

Figure S30 ^{1}H-^{1}H COSY spectrum of compound **4** in CD_3OD

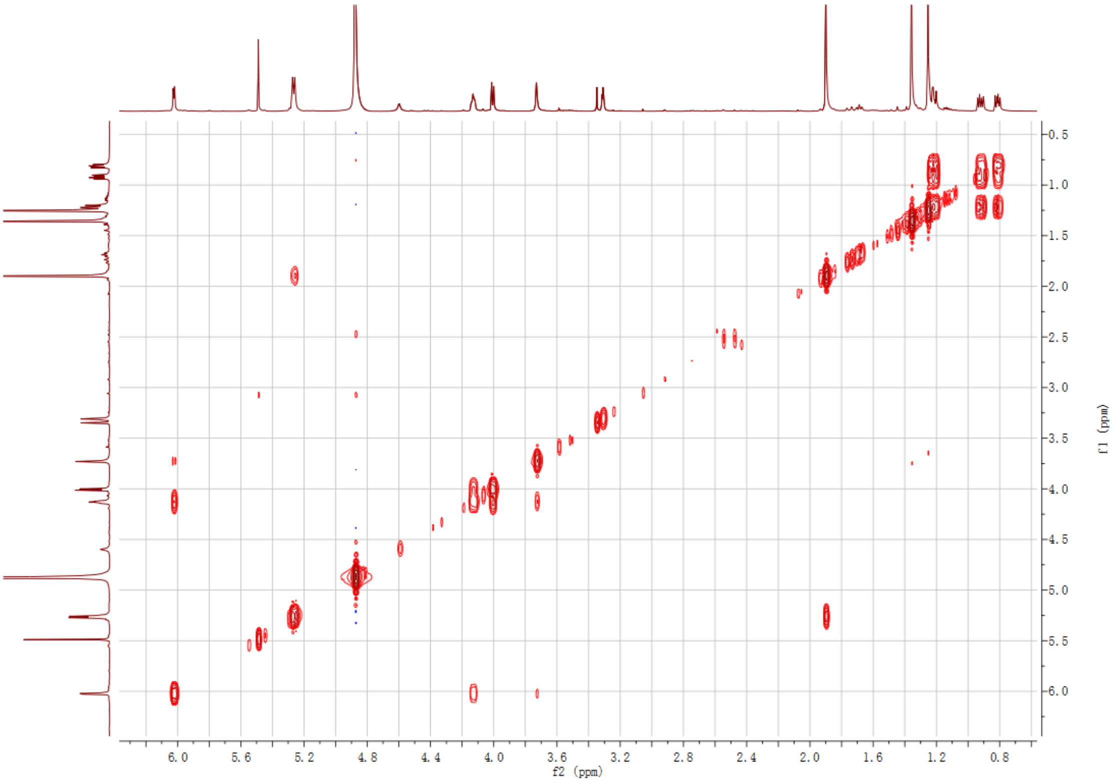

Figure S31 HSQC spectrum of compound **4** in CD_3OD

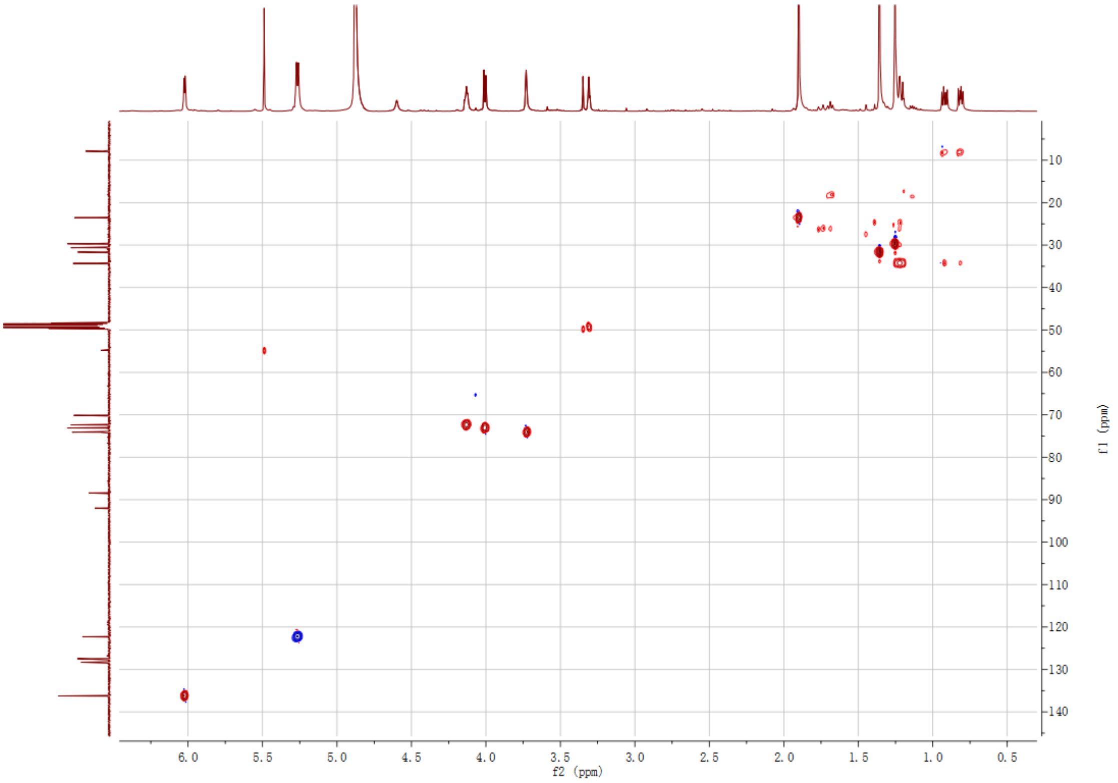

Figure S32 HMBC spectrum of compound **4** in CD_3OD

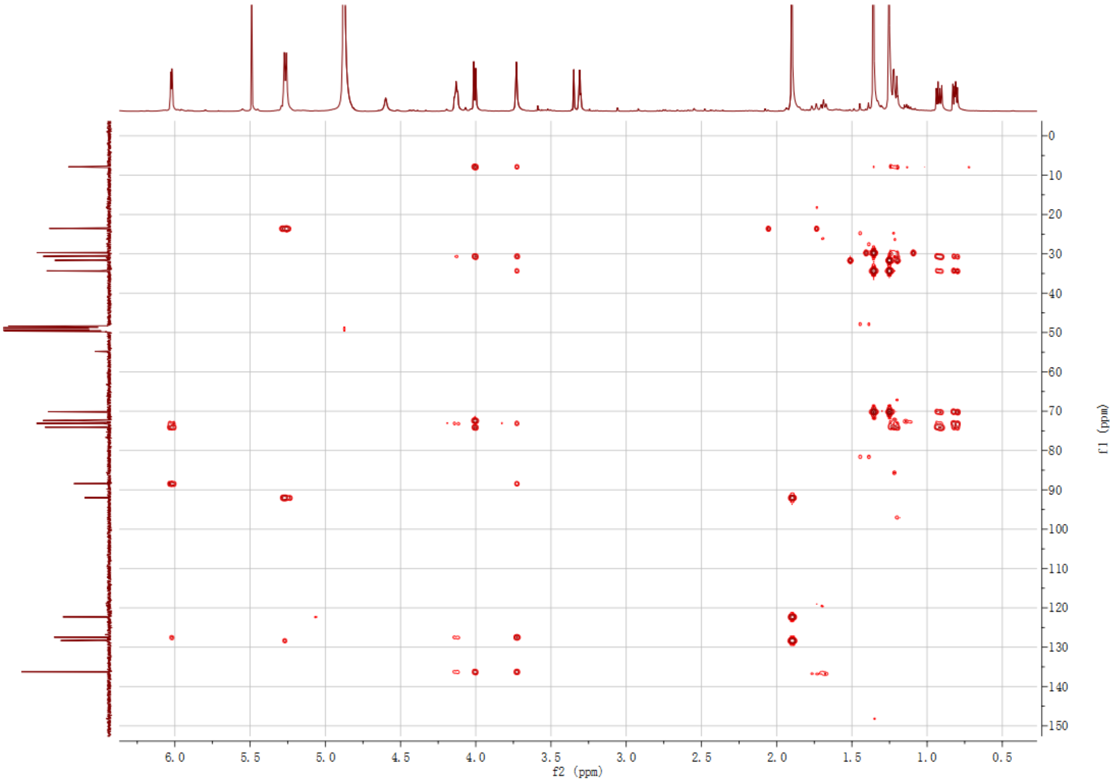

Figure S33 NOESY spectrum of compound **4** in CD_3OD

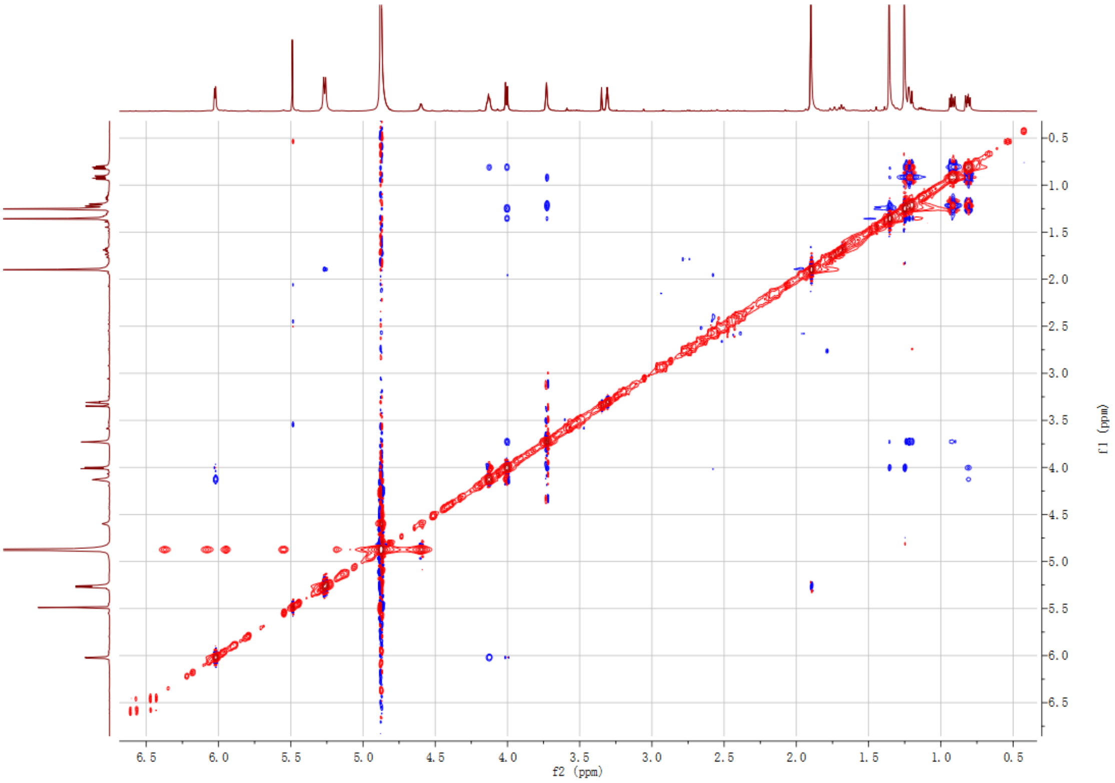

Figure S34 HR-ESI-MS spectrum of compound **4**

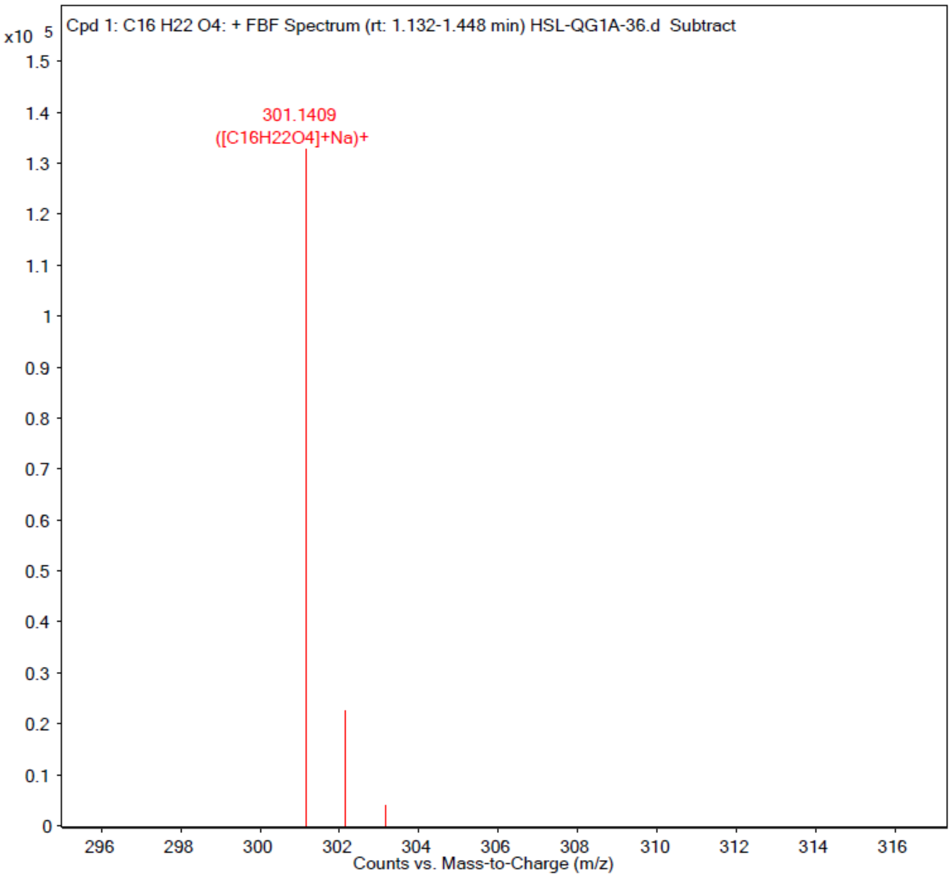

Figure S35 UV spectrum of compound **4**

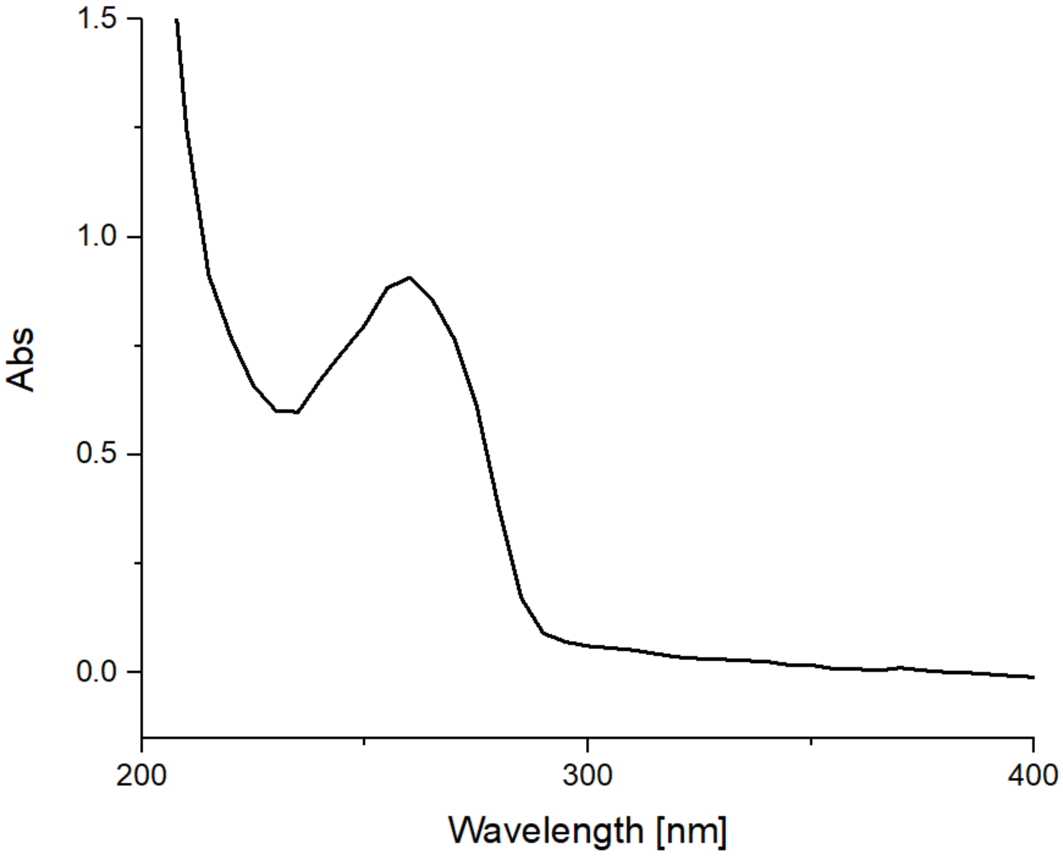

Figure S36 IR spectrum of compound **4**

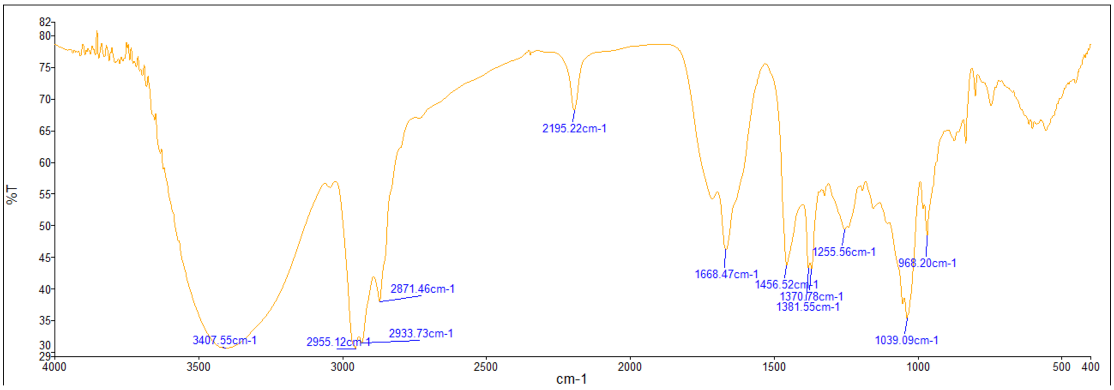

Figure S37 ^{1}H NMR spectrum of compound **5** in CD_3OD

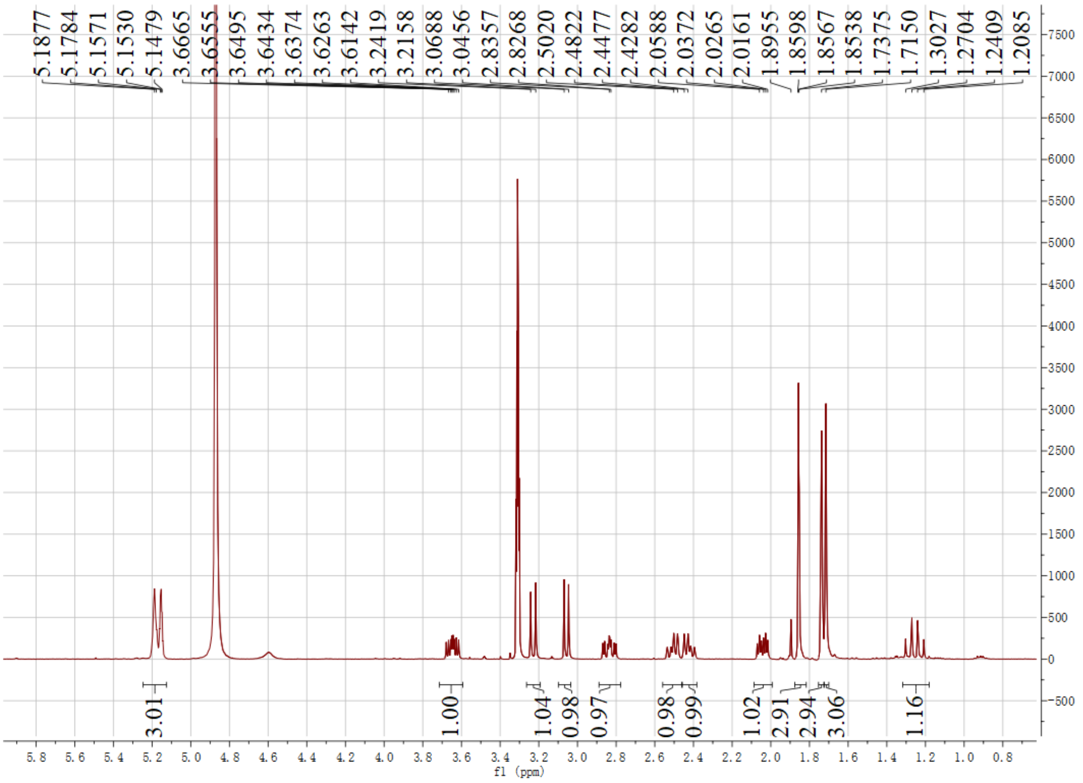

Figure S38 ^{13}C NMR spectrum of compound **5** in CD_3OD

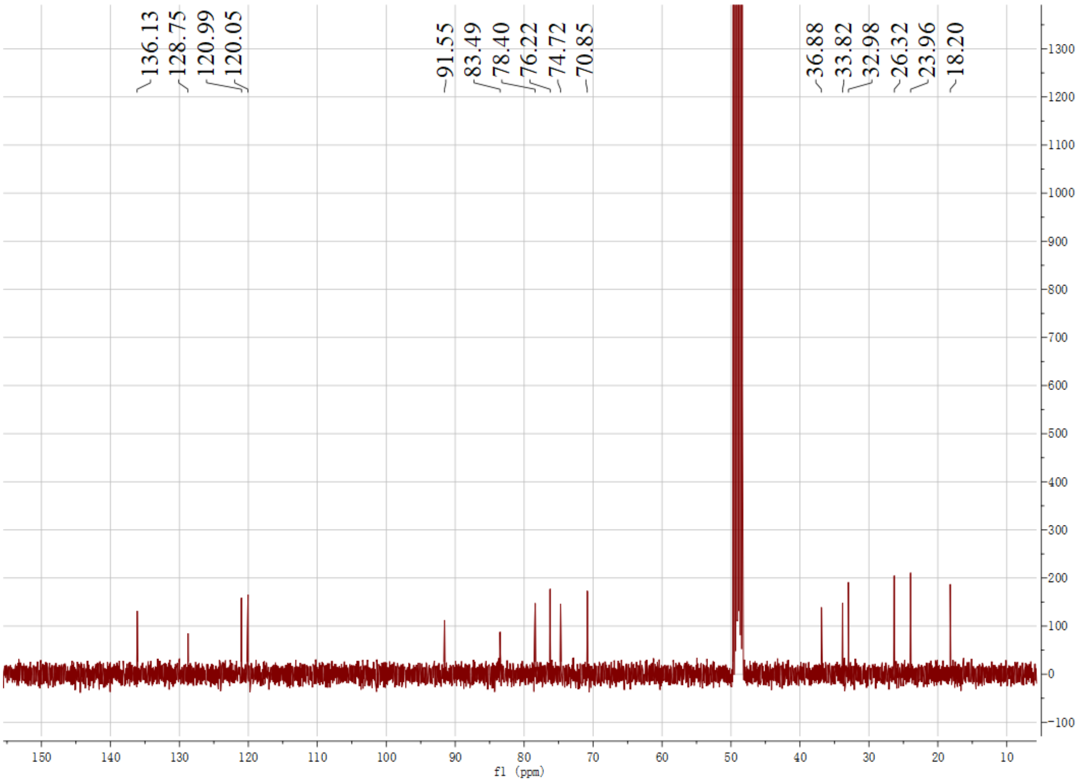

Figure S39 ^{1}H-^{1}H COSY spectrum of compound **5** in CD_3OD

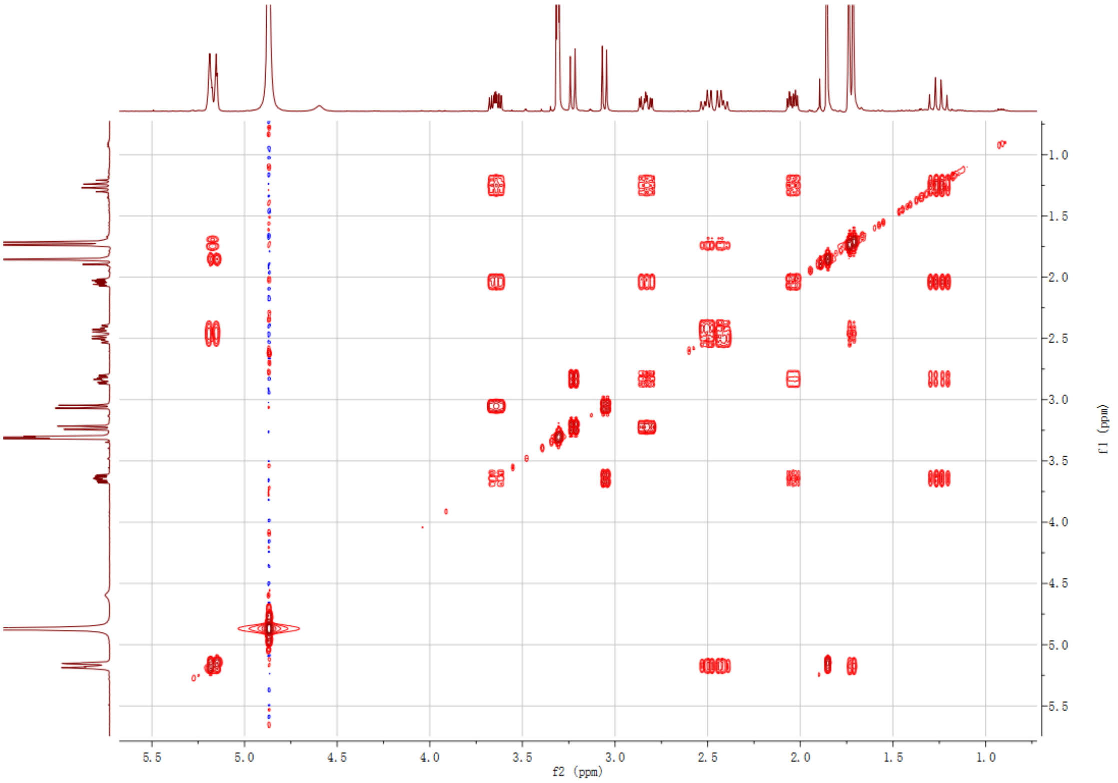

Figure S40 HSQC spectrum of compound **5** in CD_3OD

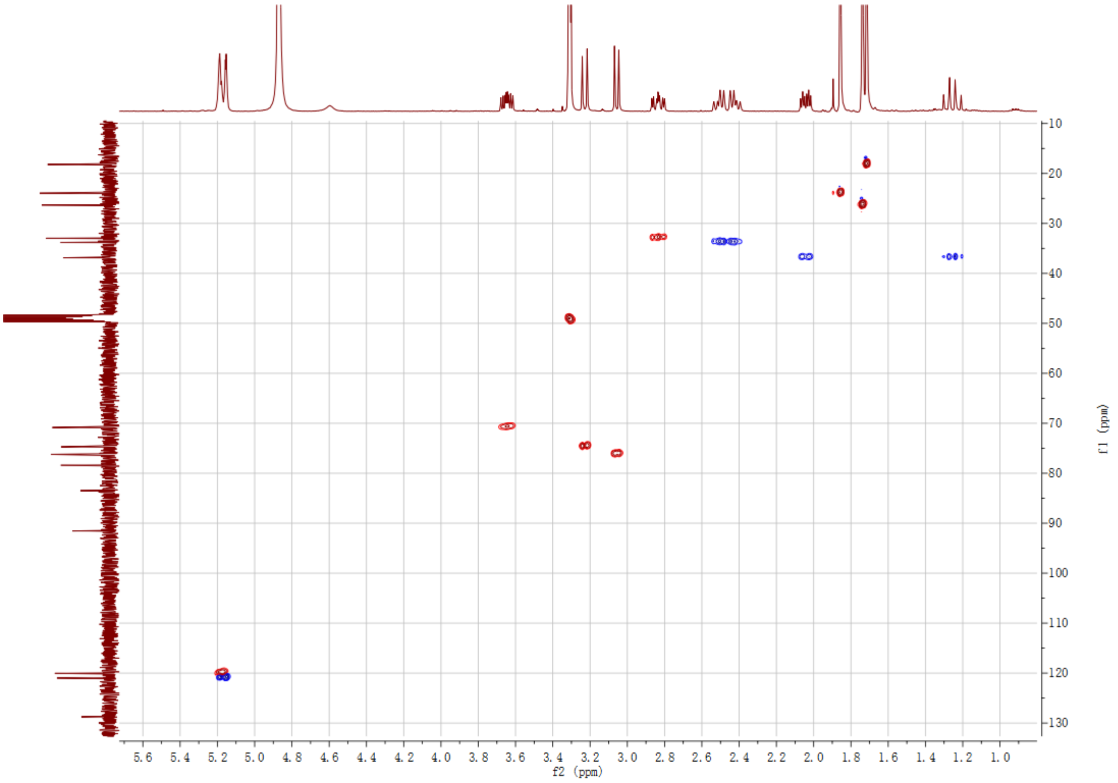

Figure S41 HMBC spectrum of compound **5** in CD_3OD

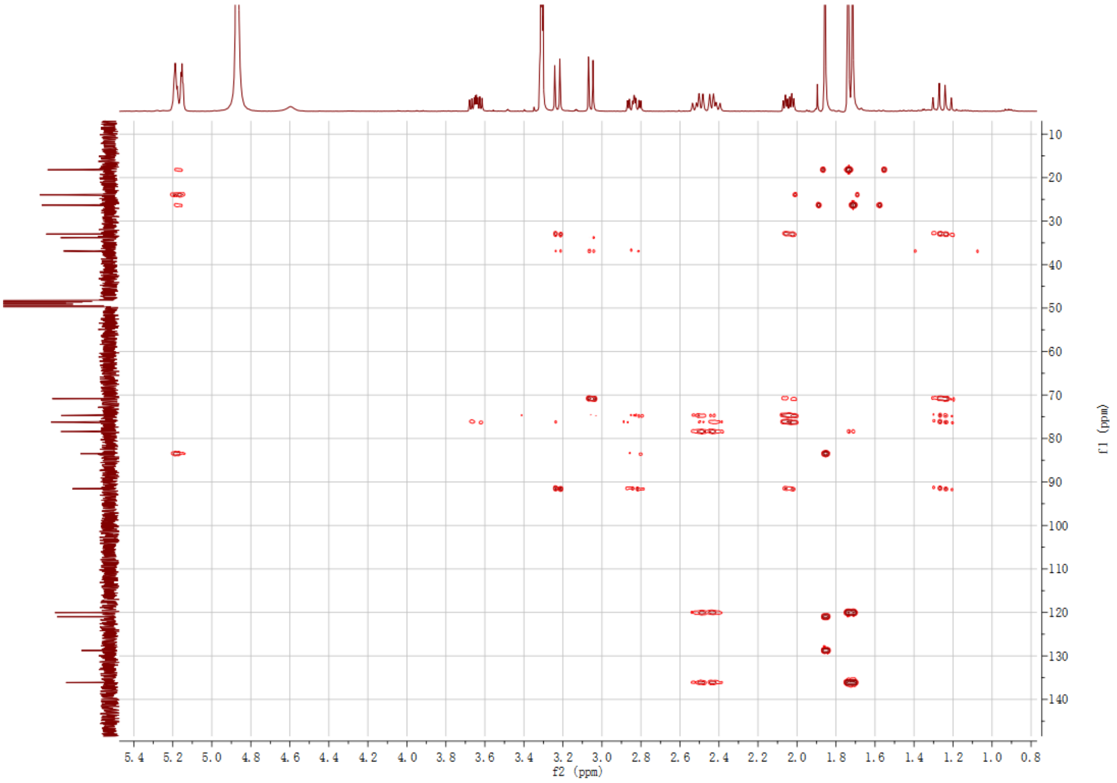

Figure S42 NOESY spectrum of compound **5** in CD_3OD

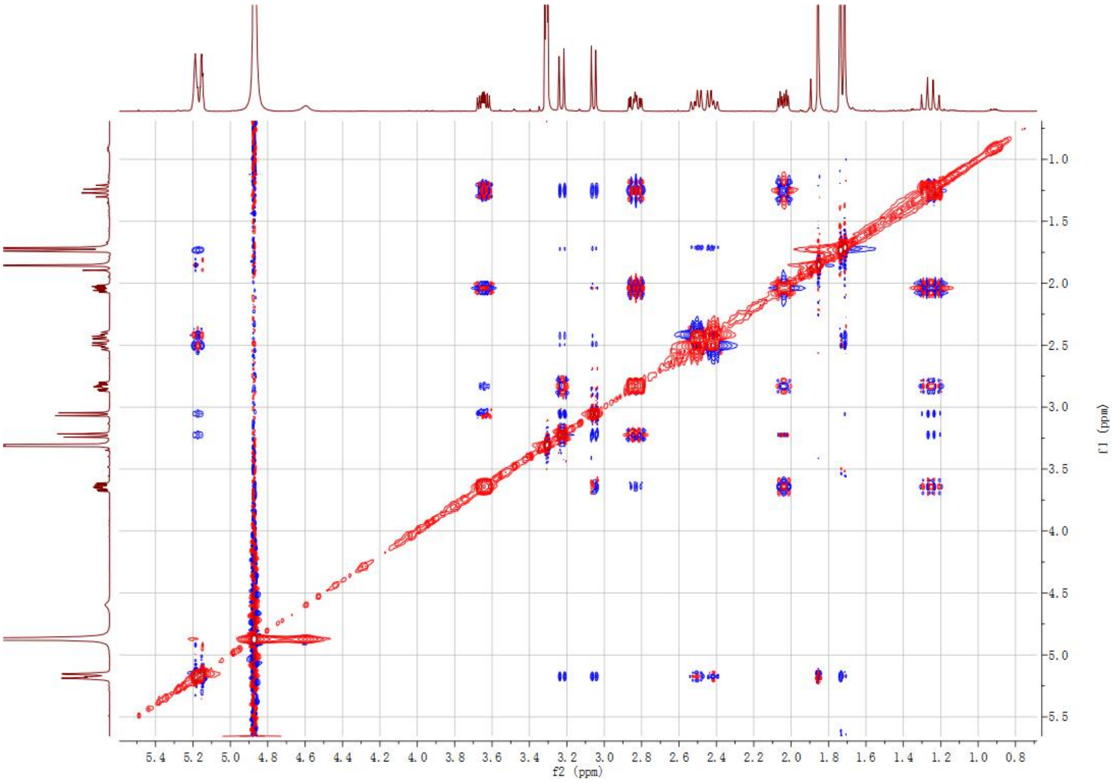

Figure S43 HR-ESI-MS spectrum of compound **5**

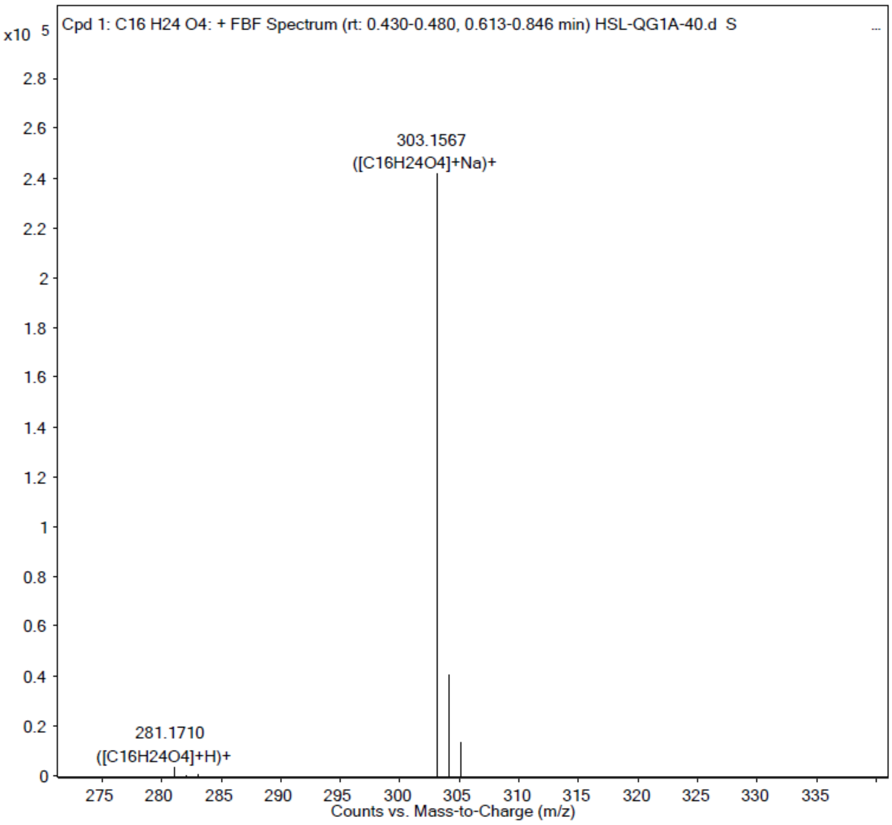

Figure S44 UV spectrum of compound **5**

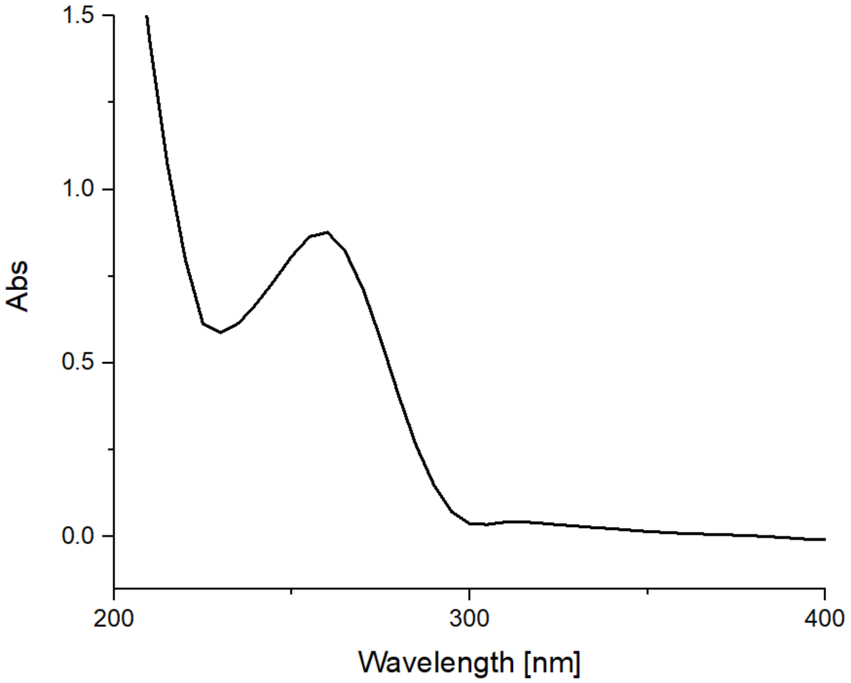

Figure S45 IR spectrum of compound **5**

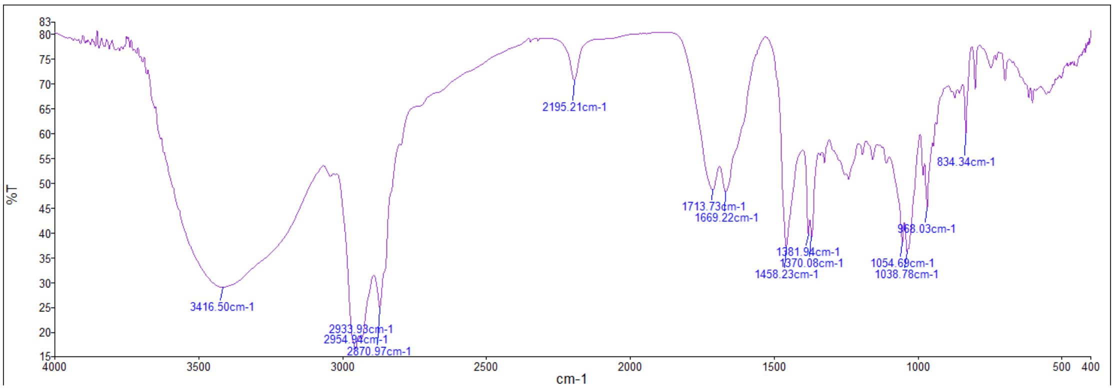

Computational Section

ECD calculation

Conformational analysis was initially performed using Confab at MMFF94 force field for new compound. Room-temperature equilibrium populations were calculated according to Boltzmann distribution law. The conformers with Boltzmann-population from above 1% were chosen for ECD calculations. The chosen conformer was optimized at B3LYP/6-311G** using Density functional theory (DFT). Then, it was further optimized in gas phase using the CPCM polarizable conductor calculation model. The theoretical calculation of ECD was conducted using Time-dependent DFT (TD-DFT) method at B3LYP/6-311G* in methanol. Rotatory strengths for total excited states were calculated. The ECD spectrum is simulated in SpecDis by overlapping Gaussian functions for each transition according to:

$$\Delta\varepsilon(E) = \frac{1}{2.297\times10^{-39}} \times \frac{1}{\sqrt{2\pi\sigma}} \sum_{i}^{A} \Delta E_i R_i e^{-\left(\frac{E-E_i}{2\sigma}\right)^2}$$

where σ represents the width of the band at 1/*e* height, and ΔE_i and R_i are the excitation energies and rotatory strengths for transition *i*, respectively. σ = 0.2-0.3 eV and UV-

Shift = 5 nm and $R^{velocity}$ have been used in this work.

Table S1. Energies of the dominative conformers of compounds **1-5.**

Compounds	No	Structure	E (Hartree)	Population (%)
(1*R*, 2*S*, 3*R*, 4*R*, 13*R*)-**1**	1	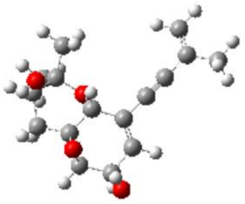	-922.53787494	70.79
	2	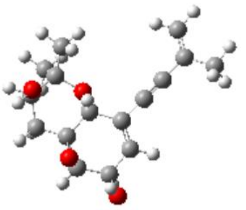	-922.53685018	29.21
(1*R*, 2*S*, 3*R*, 4*R*, 13*R*)-**2**	1	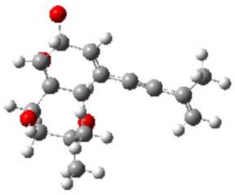	-922.53356696	56.25
	2	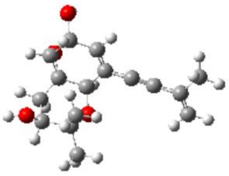	-922.53351014	43.75
(1*R*, 2*R*, 3*S*, 4*R*, 13*R*)-**3**	1	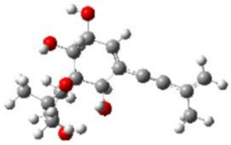	-999.02684494	56.03
	2	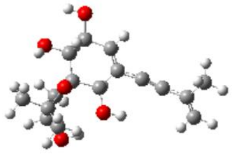	-999.02543941	43.97
(1*R*, 2*S*, 3*R*, 4*S*, 12*R*)-**4**	1	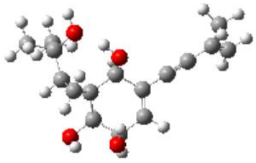	-923.74831245	54.12

	2	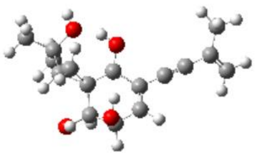	-923.74788993	39.44
	3	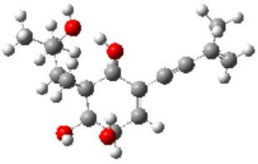	-923.74520715	6.43
(1*S*, 2*S*, 3*R*, 4*S*, 6*S*)-**5**	1	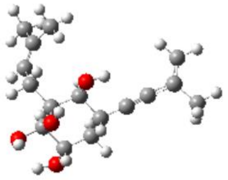	-924.97692936	20.84
	2	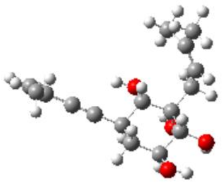	-924.97677915	20.76
	3	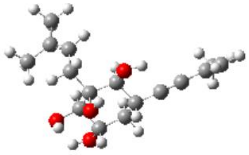	-924.97666752	29.42
	4	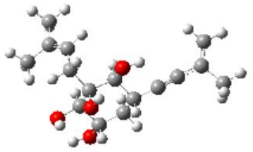	-924.97722919	28.98

^{13}C NMR calculation

The ^{13}C NMR calculation of **3a** and **3b** were calculated with the GIAO method at the MPW1PW91/6-311+G(d,p) level with PCM. The shielding constants (σ) were converted into chemical shifts (*δ*) by referencing to TMS at 0 ppm (*δ*cal = *σ*TMS – *σ*cal), where the *σ*TMS was the shielding constant of TMS calculated at the same level. The calculated chemical shifts were directly performed statistical analyses with experimental chemical shifts by using DP4+ method. The DP4+ probability, and linear correlation coefficients (R^2), were adopted for evaluation of the results.

Figure S46 Conformations of low-energy conformers of structures 3a and 3b.

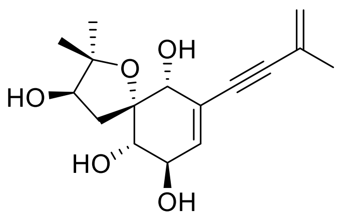

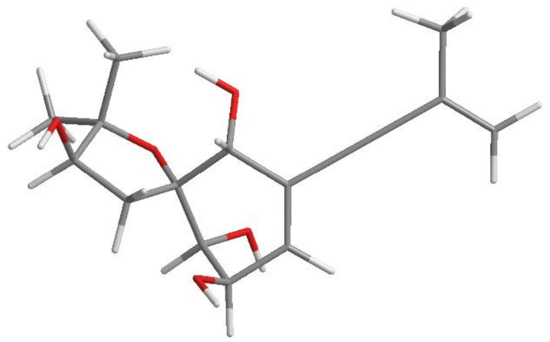

1*R*, 2*R*, 3*S*, 4*R*, 13*R*

3a

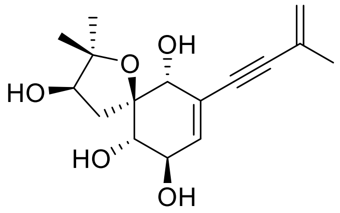

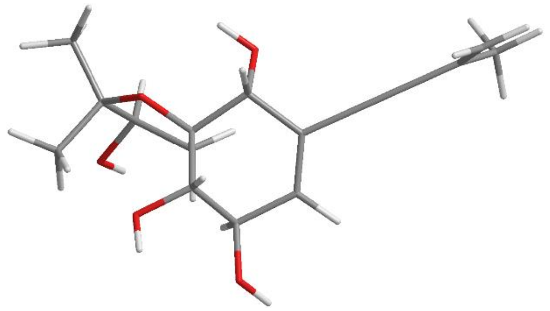

1*R*, 2*S*, 3*S*, 4*R*, 13*R*

3b

Table S2 DP4+ analysis result table of model compounds **3a** and **3b.**

NO	Experimental	**3a**	**3b**
1	135.6	139.9442	139.6799
2	128.3	129.6029	129.5934
3	126.9	126.296	126.5934
4	122.5	122.8981	125.6672
5	92.4	95.1603	95.5666
6	89.8	88.5949	87.1109
7	87.1	86.4094	86.9531
8	86.5	85.6815	86.8295
9	78.8	76.2249	76.3838
10	76.9	75.9158	73.4216
11	74	71.5636	70.2743
12	73	71.7203	68.385
13	33.7	33.2928	29.5577
14	28.3	23.137	20.9278
15	23.8	19.9035	19.7076
16	23.5	16.9438	16.3642

Figure S47 DP4+ probability analysis result.

A	B	C	D	E	F	G	H
Functional		Solvent?		Basis Set		Type of Data	
mPW1PW91		PCM		6-311+G(d, p)		Unscaled Shifts	
		DP4+	100.00%	0.00%	-	-	-
Nuclei	sp2?	Experimental	Isomer 1	Isomer 2	Isomer 3	Isomer 4	Isomer 5
C	x	135.6	139.9442	139.6799			
C	x	128.3	129.6029	129.5934			
C	x	126.9	126.296	126.5934			
C	x	122.5	122.8981	125.6672			
C		92.4	95.1603	95.5666			
C		89.8	88.5949	87.1109			
C		87.1	86.4094	86.9531			
C		86.5	85.6815	86.8295			
C		78.8	76.2249	76.3838			
C		76.9	75.9158	73.4216			
C		74	71.5636	70.2743			
C		73	71.7203	68.385			
C		33.7	33.2928	29.5577			
C		28.3	23.137	20.9278			
C		23.8	19.9035	19.7076			
C		23.5	16.9438	16.3642			

Figure S48 Regression analyses of experimental versus calculated 13C NMR chemical shifts of model compounds **3a** and **3b**

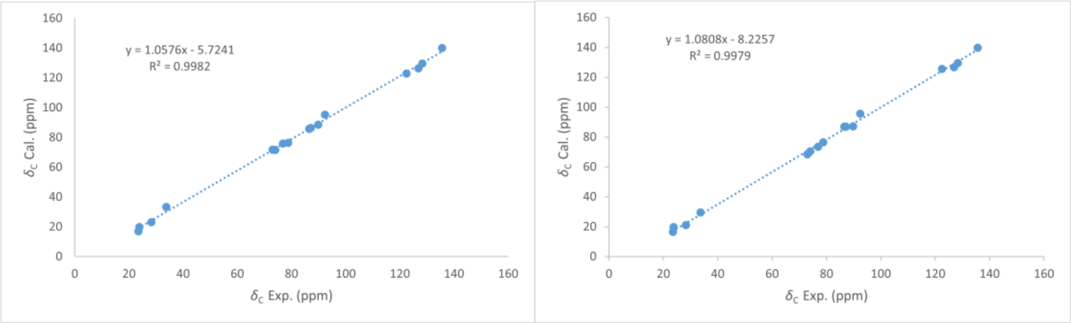

Table S3. ^{1}H (400 MHz) and ^{13}C (100 MHz) NMR, and COSY, HMBC and NOESY assignment of **1** in CD_3OD.

No	**1**				
	δ_C, type	δ_H (J in Hz)	COSY	HMBC	NOESY
1	67.8, CH	4.46, t (2.0)		2, 5, 6, 7, 14	12*α*, 15
2	61.0, C				
3	60.1, CH	3.26, t (1.8)	4	12	12*β*
4	64.7, CH	4.41, dt (5.3, 1.8)	3, 5	2, 6	
5	133.2, CH	5.93, dt (5.3, 2.0)	4	1, 7	
6	123.3, C				
7	86.8, C				
8	93.3, C				
9	128.3, C				

10	122.3, CH_2	5.26, m		8, 9, 11	
11	23.5, CH_3	1.89, m		8, 9, 10	
12α	36.3, CH_2	2.27, dd (13.3, 12.0)	13	2, 3	1, 15
12β	36.3, CH_2	1.57, dd (13.3, 5.0)	13	2, 3	3
13	73.2, CH	3.70, dd (12.0, 5.0)	12	14, 15	16
14	78.6, C				
15	16.5, CH_3	1.30, s		13	1, 12α
16	28.2, CH_3	1.33, s		1, 14	13

Table S4. ^{1}H (400 MHz) and ^{13}C (100 MHz) NMR, and COSY, HMBC and NOESY assignment of **2** in CD_3OD.

No	**2**				
	δ_C, type	δ_H (J in Hz)	COSY	HMBC	NOESY
1	68.4, CH	4.42, t (2.4)		2, 5, 6, 7, 14	12α, 15
2	57.4, C				
3	58.6, CH	3.26, t (2.3)	4	2, 12	12β
4	66.9, CH	4.46, dd (4.5, 2.3)	3, 5	6	
5	134.7, CH	5.73, dd (4.5, 2.4)	4	1, 6, 7	
6	121.6, C				
7	86.7, C				
8	92.3, C				
9	128.5, C				
10	121.9, CH_2	5.24, m		8, 9, 11	
11	23.5, CH_3	1.88, m		8, 9	
12α	34.9, CH_2	2.72, dd (14.8, 3.3)	13	2, 3, 14	1, 15
12β	34.9, CH_2	1.55, dd (14.8, 2.7)	13	2, 3, 14	3
13	72.4, CH	3.62, dd (3.3, 2.7)	12	14	15
14	78.0, C				
15	26.4, CH_3	1.32, s		14	1, 12α, 13
16	22.3, CH_3	1.37, s		14	

Table S5. ^{1}H (400 MHz) and ^{13}C (100 MHz) NMR, and COSY, HMBC and NOESY assignment of **3** in CD_3OD.

No	**3**				
	δ_C, type	δ_H (J in Hz)	COSY	HMBC	NOESY
1	74.0, CH	4.04, t (2.5)		2, 5, 6, 12	3, 12β
2	89.8, C				
3	76.9, CH	3.51, d (8.1)	4	1, 2, 12	1
4	73.0, CH	3.97, dt (8.1, 2.8)	3, 5	2	12α, 13, 15
5	135. 6, CH	5.85, dd (2.8, 2.5)	4	1, 6, 7	
6	126.9, C				
7	87.1, C				
8	92.4, C				
9	128.3, C				

10	122.5, CH_2	5.28, m		8, 9	
11	23.5, CH_3	1.90, m		8, 9, 10	
12*α*	33.7, CH_2	1.79, dd (13.9, 1.8)	13	2, 14	4
12*β*	33.7, CH_2	2.48, dd (13.9, 6.3)	13	2, 14	1, 16
13	78.8, CH	3.87, dd (6.3, 1.8)	12	2, 14	4
14	86.5, C				
15	28.3, CH_3	1.26, s		13, 14, 16	4
16	23.8, CH_3	1.27, s		14	12*β*

Table S6. ^{1}H (400 MHz) and ^{13}C (100 MHz) NMR, and COSY, HMBC and NOESY assignment of **4** in CD_3OD.

No	**4**				
	δ_C, type	δ_H (*J* in Hz)	COSY	HMBC	NOESY
1	74.1, CH	3.73, s		2, 3, 6, 7	3, 13
2	30.6, C				
3	73.1, CH	4.01, d (5.2)	4	1, 12	1
4	72.4, CH	4.13, m	3, 5		
5	136.2, CH	6.02, d (3.2)	4	1, 6, 7	
6	127.5, C				
7	88.4, C				
8	92.0, C				
9	128.3, C				
10	122.3, CH_2	5.26, m		8, 9, 11	
11	23.6, CH_3	1.90, m		9, 10	
12	7.9, CH_2	0.92, dd (9.7, 4.8)	13	2, 3	
		0.81, dd (7.5, 4.8)			
13	34.3, CH	1.21, m	12	1	1
14	70.1, C				
15	31.7, CH_3	1.36, s		13, 14	
16	29.7, CH_3	1.25, s		13, 14	

Table S7. ^{1}H (400 MHz) and ^{13}C (100 MHz) NMR, and COSY, HMBC and NOESY assignment of **5** in CD_3OD.

No	**5**				
	δ_C, type	δ_H (*J* in Hz)	COSY	HMBC	NOESY
1	74.7, CH	3.23, d (10.5)	6	8	3, 5*β*, 12*β*
2	78.4, C				
3	76.2, CH	3.06, d (9.3)	4	12	1, 5*β*
4	70.9, CH	3.65, m	3, 5		6
5*α*	36.9, CH_2	2.04, dt (13.2, 4.6)	4, 6		
5*β*	36.9, CH_2	1.26, m	4, 6		1, 3
6	33.0, CH	2.83, m	5, 1	7, 8	4
7	83.5, C				
8	91.6, C				

9	128.8, C				
10	121.0, CH_2	5.19, m		7, 11	
		5.15, m			
11	24.0, CH_3	1.86 m		9, 10	
12*α*	33.8, CH_2	2.51, dd (13.4, 7.9)	13	1, 2, 3	
12*β*	33.8, CH_2	2.42, dd (13.4, 7.8)	13	1, 2, 3	1
13	120.1, CH	5.18, m	12	15, 16	
14	136.1, C				
15	26.3, CH_3	1.74, s		13, 14, 16	
16	18.2, CH_3	1.71, s		13, 14, 15	

Table S8. ^{1}H (400 MHz) and ^{13}C (100 MHz) NMR of **6-8** in CD_3OD.

No	**6**		**7**		**8**	
	δ_C	δ_H (*J* in Hz)	δ_C	δ_H (*J* in Hz)	δ_C	δ_H (*J* in Hz)
1	68.3	4.60, t (1.9)	78.0	4.25, s	67.6	4.17, t (2.0)
2	58.1		32.2		63.3	
3	57.5	3.14, t (2.1)	75.3	3.44, m	60.4	3.34, overlapped
4	64.9	4.44, dt (5.5, 1.9)	69.2	4.14, dd (5.2, 1.1)	66.2	4.43, dd (5.0, 2.5)
5	133.0	5.93, dt (5.5, 2.2)	134.4	6.10, d (5.2)	134.3	5.76, dd (4.5, 2.5)
6	123.8		129.4		124.5	
7	86.9		88.2		87.6	
8	93.4		92.5		91.6	
9	128.4		128.4		128.4	
10	122.2	5.24, s	122.3	5.28, dd (3.8, 3.0)	122.2	5.28, m; 5.25, m
				5.26, m		
11	23.5	1.89, t (1.1)	23.6	1.90, d (1.1)	23.5	1.90, m
12	34.8	2.71, dd (14.8, 3.3)	12.3	0.69, m	32.2	2.93, dd (14.7, 8.9)
		1.55, dd (14.8, 2.9)				2.16, dd (14.7, 6.2)
13	72.6	3.64, t (2.9)	33.5	1.69, dd (7.8, 4.6)	118.9	5.13, m
14	78.1		83.9		136.7	
15	22.3	1.39, s	28.5	1.37, s	18.1	1.69, s
16	26.4	1.32, s	26.0	1.20, s	26.0	1.74, s
